# Interleukin-33 (IL-33) promotes DNA damage-resistance in lung cancer

**DOI:** 10.1038/s41419-025-07624-x

**Published:** 2025-04-11

**Authors:** Haoge Luo, Liping Liu, Xiaoping Liu, Yingdong Xie, Xin Huang, Ming Yang, Chen Shao, Dong Li

**Affiliations:** 1 Department of Immunology, College of Basic Medical Sciences, Jilin University, Changchun, China; 2 Department of Pathophysiology, College of Basic Medical Sciences, Jilin University, Changchun, China; 3 Department of Microbiology, College of Basic Medical Sciences, Jilin University, Changchun, China; 4 Department of Biochemistry & Molecular Biology, College of Basic Medical Sciences, Jilin University, Changchun, China; 5 Medical Basic Research Innovation Center of Airway Disease in North China, Key Laboratory of Pathobiology, Ministry of Education, Jilin University, Changchun, China

**Keywords:** Interleukins, Cell death and immune response, Cancer microenvironment, Extracellular signalling molecules

## Abstract

Resistance to DNA damage is one of the primary mechanisms by which tumor cells evade the effects of standard chemotherapeutic agents and radiotherapy. Dynamic and complex interactions between the tumor microenvironment (TME) and tumor cells critically influence the DNA damage response. Interleukin-33 (IL-33) is a multifunctional cytokine secreted at high levels in response to cellular damage and stress. Recently, increasing evidence has suggested that IL-33 plays a key role in promoting the therapeutic resistance of tumors. However, the actual source of IL-33 during cancer therapy and how IL-33 contributes to a resistant TME remain incompletely understood. In this study, we found that both cancer-associated fibroblasts (CAFs) and tumor cells treated with DNA damage-inducing agents expressed and secreted high levels of IL-33, subsequently leading to enhanced DNA damage repair efficacy. Mechanistically, nuclear IL-33 primarily functions as a transcriptional co-activator of homologous recombination repair (HRR) genes, whereas the active form of IL-33 can drive the non-homologous end joining (NHEJ) pathway via the canonical IL-33/ST2 axis. Overall, we demonstrated that IL-33 plays a key role in mediating a DNA damage-resistant TME, which could represent a potential therapeutic vulnerability in chemoresistant cancer cells.

## Introduction

Accumulating evidence suggests that the complex interplay among diverse cell types within tumors can dramatically impact the therapeutic outcomes of standard chemotherapeutic agents and radiotherapy [1, 2]. These treatments primarily function by inducing DNA lesions that hinder cancer cell replication and promote apoptosis. However, under the influence of surrounding environment, cancer cells can develop a range of resistance mechanisms, enabling them to survive and proliferate despite therapeutic interventions [3, 4]. Elucidating how cancer cells establish DNA damage-resistance is critical for the development of more effective and targeted treatments. Solid tumors comprise various non-cancerous cells, including vascular endothelial cells, fibroblasts, and immune cells, which actively interact with factors such as the extracellular matrix, oxygen, chemokines, and cytokines to shape the tumor microenvironment (TME) [5, 6]. Therein, cytokines and cytokine receptors have been demonstrated to play paradoxical roles in tumor progression over the past several decades. Depending on the balance between the pro- and anti-inflammatory TME, cytokines can mediate an antitumor response by driving the death of abnormal cells, but during chronic conditions, they can also facilitate malignancy, metastasis, and chemoresistance [7–9]. Given the key role of cytokines in the TME, many of which are dysregulated in various types of cancer, targeting cytokine networks to enhance the efficacy of other treatments may represent a promising therapeutic strategy [10, 11].

Interleukin-33 (IL-33) belongs to the IL-1 family of cytokines and is highly expressed in the nuclei of epithelial, endothelial, and fibroblast-like cells [12]. Full-length human IL-33(FL-IL-33) contains three domains: the N-terminal nuclear domain, the central protease sensor domain that harbors cleavage sites for allergens and inflammatory proteases, and the C-terminal cytokine domain [13]. Upon cellular stress and damage, IL-33 is quickly released into the cytoplasm to exert its primary role as an alarmin by binding to its specific receptor, suppression of tumorigenicity 2 (ST2). During this process, FL-IL-33 is cleaved by proteases released by immune cells, leading to the formation of a shortened, mature form of IL-33 that is more bioactive than FL-IL-33 [14, 15]. The mature form of IL-33 lacks the N-terminal nuclear domain but can function as an IL-1-like cytokine and trigger Myd88-dependent signaling pathways in cells expressing the ST2 receptor [16]. Although the function of IL-33 in driving the immune response has been extensively examined, its role in regulating the therapeutic resistance of tumor cells is only beginning to be explored. Previous studies have found that the IL-33/ST2 axis has tumor-promoting effects in cancer cells [17, 18]. Remarkably, IL-33 is involved in Fluorouracil (5-Fu) resistance in murine melanoma cells and is associated with polyploidy, a hallmark of drug resistance and chromosome instability (CIN) [19]. Consistently, Huang et al. showed that mice with ST2 deficiency had impaired radioprotective functions, as illustrated by decreased hematopoietic stem cell regeneration after whole-body irradiation [20]. Moreover, IL-33 can also exert its anti-DNA damage role in a canonical IL-33/ST2-independent manner, as nuclear IL-33 is sufficient to promote chemoresistance, with almost no IL-33 detected in the culture medium [21]. Taken together, these findings suggest that targeting IL-33 may be beneficial for increasing the efficacy of DNA damage-inducing anticancer drugs.

One of the most abundant stromal cells surrounding cancer cells are cancer-associated fibroblasts (CAFs), which are reported to be actively involved in cell growth, invasion, and treatment resistance by secreting several crucial cytokines, chemokines and exosomal miRNAs [22, 23]. CAFs not only mediate drug resistance through their secretory factors but may also serve as a physical barrier to hinder the delivery of anticancer drugs as well as immune cell infiltration during cancer treatment [24]. Importantly, upon cellular stress, such as chemotherapy or radiotherapy, the secretory functions of CAFs are significantly enhanced, affecting multiple stages of cancer progression [25, 26]. Thus, better deciphering CAFs and their interacting signaling pathways will be crucial for optimizing the sensitivity of anticancer therapies. Notably, CAF-derived IL-33 was demonstrated to promote breast cancer lung metastasis by recruiting neutrophils and inflammatory monocytes to the metastatic TME [27]. Consistently, hyperactivation of IL-33/ST2 in CAFs has also been shown to promote the invasion and metastasis of ovarian cancer [28]. However, whether and how IL-33-expressing CAFs contribute to chemoresistance remain unclear.

In this study, we investigated the specific role of IL-33 from both CAFs and tumor cells in regulating the chemosensitivity of lung cancer. First, we found that CAFs isolated from lung cancer patients expressed high levels of IL-33, which could dramatically promote chemoresistance of cancer cells upon co-culturing. In addition, we showed that nuclear and the active forms of IL-33 (IL-33 cytokine domain) can promote cellular DNA damage repair through different mechanisms. Nuclear IL-33 primarily functions as a transcriptional co-activator of homologous recombination repair (HRR) genes, whereas the active form of IL-33 can drive the non-homologous end joining (NHEJ) pathway via the canonical IL-33/ST2 axis. In short, we demonstrated that during cancer chemotherapy, IL-33 facilitates a DNA damage-resistant TME through different mechanisms.

## Materials and methods

### Chemicals

Doxorubicin was purchased from TargetMol (Boston, MA, USA), whereas cisplatin and puromycin were obtained from MedChem Express (Shanghai, China). FR 180204 and SB 203580 were purchased from Beyotime (Shanghai, China). Human IL-33 recombinant protein (PeproTech®) was obtained from Thermo Fisher Scientific.

### Cell culture, virus infection, plasmid transfection

A549 and H1299 cells were obtained from the American Type Culture Collection (ATCC, RRID: CVCL_0023 and RRID: CVCL_B7N8) and A549-DDP cells were obtained from Guangzhou Cellcook Biotech Co., Ltd. The cells were cultured in DMEM (Gibco) supplemented with 10% fetal bovine serum and 100 U/mL penicillin-streptomycin. Lung cancer-associated fibroblasts (CP-H245) and normal lung-associated fibroblasts (CP-H011), along with their respective cultures (CAFs: CM-H245, NAFs: CM-H011), were purchased from Pricella Life Science & Technology. All experiments involving CAFs and NAFs were conducted using cells within the first 2–5 passages. The Cells were maintained in a 5% CO_2_ atmosphere at 37 °C. To generate cell lines stably expressing IL-33-F or IL-33-C, lentiviral particles were synthesized using GENECHEM. The cDNAs for human IL-33-F (NM_033439) and IL-33-C were subcloned into the GV348 vector. Cells were infected with lentivirus and selected with 2 μg/mL puromycin for stable expression. Plasmid transfection was performed using OPTI-MEM (Gibco, Germany) and Lipofectamine 2000 (Invitrogen, Carlsbad, CA, USA), according to the manufacturer’s protocol. pDRGFP (RRID:Addgene_26475), pCBAScel (RRID:Addgene_26477), and pimEJ5GFP (RRID:Addgene_44026) plasmids were obtained from Addgene.

### Lentivirus infection

To generate cells stably expressing ST2 shRNA, lentiviral particles were synthesized by Gene Pharma (Shanghai, China), and infected cells were selected using G418 (Sigma, A1720-1G) according to the recommended protocol. The targeting sequences for ST2 knockdown are as follows: shST2#1 5’-GCACCTCTTGAGTGGTTTAAG-3’; shST2#2 5’-GCTAAACCTTACAAGACTAGG-3’; shST2#3 5’-GCACTTTGTTCACCAGATTCT-3’.

### Cell viability assay

Cell viability was assessed using the 3-(4,5-dimethylthiazol-2-yl)-2,5-diphenyl tetrazolium bromide) reduction assay. Cells were seeded at a density of 8000 cells per well in 96-well plates at a final volume of 100 μL and then treated with serial drug dilutions for 72 h. After treatment, the medium was removed and 5 mg/mL MTT solution was added to each well and incubated for 4 h. The formazan crystals formed were dissolved in 150 µL of DMSO and the absorbance was measured at 490 nm.

### Quantitative Real-Time-PCR (qPCR)

Total RNA was isolated from the cells using TRIzol reagent (Invitrogen) according to the manufacturer’s protocol. cDNA was synthesized from 2 μg of total RNA using the Hifair® III 1st Strand cDNA Synthesis SuperMix for qPCR (YEASEN). Real-time PCR was performed on a Bio-Rad CFX96 machine using Hieff® qPCR SYBR Green Master Mix (YEASEN). All the PCR primer sequences used are listed in Table S1.

### Comet assay

An alkaline comet assay was performed using a comet assay kit (ab238544; Abcam), according to the manufacturer’s recommended protocols. Briefly, lung cancer cells were collected after drug treatment and single-cell suspensions were prepared. The cells were mixed with low-melting agarose, coated onto glass slides, and immersed in lysis solution. Subsequently, gel electrophoresis and slide neutralization were performed. The tail moment and DNA damage were analyzed using the CASPlab (RRID:SCR_007249) software.

### RNA-seq analysis

Cells infected with the virus were seeded at a density of 2 × 10^6^ cells per 10 cm dish and incubated overnight. After drug treatment, TRIzol reagent was used for cell lysis according to the manufacturer’s instructions. Three independent biological replicates were used for each experiment. The lysates were sent to Personalbio (Shanghai, China) for cDNA library construction and sequenced on a NovaSeq 6000 platform (Illumina). Sequencing data were filtered to obtain high-quality sequences using the fastp (0.22.0, RRID:SCR_016962) software and mapped to the reference genome using HISAT2 (v2.1.0, RRID:SCR_015530). HTSeq (0.9.1, RRID:SCR_005514) software was used to count the number of reads for each gene, and gene expression was normalized using Fragments Per Kilobase of transcript per million mapped reads (FPKM). Differentially expressed genes were identified using the DESeq (v1.38.3, RRID:SCR_000154) package with the criteria of |log2FoldChange | ≥ 1 and an adjusted *p* < 0.05.

### Western blotting

Cells were lysed in RIPA buffer (Beyotime) supplemented with protease inhibitors (Sigma) and phosphatase inhibitors (MedChem Express). Protein concentration was measured using the BCA assay (Thermo Scientific). Protein samples were separated by SDS-PAGE and transferred to a polyvinylidene fluoride (PVDF) membrane (Millipore). The Membranes were blocked with 5% non-fat dried milk and incubated overnight at 4 °C with primary antibodies. Subsequently, the membranes were incubated with appropriate secondary antibodies at room temperature for 2 h. Primary antibodies used included ERK (4695S, RRID:AB_390779), p-ERK (Thr202/Tyr204) (4370S, RRID:AB_2315112), p38 (8690S), p-p38 (Thr180/Tyr182) (4511S), JNK (9252S, RRID:AB_2250373), p-JNK (Thr183/Tyr185) (9255S, RRID:AB_2307321), DNA-PKcs (38168S, RRID:AB_2799128), p-DNA-PKcs (Ser2056) (68716S, RRID:AB_2939025), ATM (2873S, RRID:AB_2062659), p-ATM (Ser1981) (13050S, RRID:AB_2798100), cleaved-PARP (9541S, RRID:AB_331426), and phospho-H2AX (Ser139) (2577S, RRID:AB_2118010), all obtained from Cell Signaling Technology. The antibodies against BRCA1 (sc-6954, RRID:AB_626761) and BRCA2 (sc-293185) were purchased from Santa Cruz Biotechnology. Antibodies against β-actin (81115-1-RR, RRID:AB_2923704) and the HA-tag (51064-2-AP, RRID:AB_11042321) were obtained from Proteintech. Antibodies against ST2L (NBP1-85251, RRID:AB_11017877) and IL-33 (PA5-47006, RRID:AB_2609883) were purchased from Novus and Thermo Fisher Scientific, respectively.

### Immunofluorescence staining

The cells were fixed with 4% (w/v) paraformaldehyde in PBS for 20 min, washed three times with 1x PBS and subjected to proteinase K digestion for 1 min. Following this, The cells were permeabilized with 0.1% Triton X-100 for 7 min, blocked with 5% bovine serum albumin (BSA) for 30 min, and incubated with primary antibodies overnight at 4 °C. The next day, cells were stained with secondary antibodies for 30 min and subsequently incubated with DAPI for 5 min. Images were acquired using a FV1000 confocal laser microscope (Olympus).

### Extraction of nuclear proteins

Nuclear and cytoplasmic proteins were extracted from cells following the manufacturer’s instructions for the Nuclear and Cytoplasmic Protein Extraction Kit (Beyotime Biotechnology, Shanghai, China). The purity of the cellular fractions was determined by western blot analysis, with tubulin as a cytoplasmic marker and lamin B1 as a nuclear marker.

### Enzyme-linked immunosorbent assay (ELISA)

Cytokine concentrations in the culture media were quantitatively determined using ELISA according to the manufacturer’s instructions, including IL-33 (R&D Systems, USA). The absorbance was measured at 450 nm for each well.

### HR and NHEJ assay

NHEJ and HR reporter assays were used to evaluate the ability of cells to repair DNA double-stranded breaks. In this study, A549 overexpressing cell lines (IL-33-F and IL-33-C) and a control cell line (Ctrl) were utilized. For each experimental condition, 2 × 10^6^ cells were seeded in six-well plates. The following day, the cells were transfected with pDRGFP/pimEJ5GFP and pCBASceI using Lipofectamine 2000, according to the manufacturer’s protocol. After 72 h, the cells were harvested, washed with PBS, and analyzed using an Accuri C6 Flow Cytometer (BD Biosciences, Franklin Lakes, NJ, USA) to determine the percentage of GFP-positive cells.

### Colony formation assay

Cells were seeded at a density of 1500 cells per well in six-well plates and cultured in medium containing the indicated drugs for 14 days, with the medium refreshed every 2 days. Cells were fixed with 4% paraformaldehyde and stained with 0.05% crystal violet.

### Human lung cancer sample and spatial transcriptomics

The surgically resected lung cancer tissue (71 years old, invasive adenocarcinoma, moderately differentiated) was immediately submerged in cold PBS and cut into ~5 mm pieces. The tissue sample was placed in an OCT-filled mold and snap-frozen on dry ice. Spatial transcriptome sequencing was performed using the Visium Technology Platform from 10x Genomics at Shanghai Majorbio Bio-Pharm Technology Co., Ltd.

### Cleavage Under Targets and Tagmentation (CUT&Tag)

Cells infected with the virus were seeded at a density of 2 × 10^7^ cells per 10 cm dish and incubated overnight. The cells were sent to Jingjie PTM BioLab Co., Inc. (Hangzhou, China) for CUT&Tag analysis. Briefly, the CUT&Tag assay was performed using the NovoNGS CUT&Tag 4.0 High-Sensitivity Kit (Illumina), according to the manufacturer’s protocol. Library quality was assessed on the Agilent 5400 system (Agilent, USA) and quantified by qPCR. Raw data in fastq format were processed using the Trimmomatic (RRID:SCR_011848) software. All downstream analyses were based on high-quality clean data. The bam file generated from uniquely mapped reads was used as input for peak calling with macs2 software, applying a cutoff q-value < 0.05. The peaks were annotated using in-house scripts. Peak analysis involved counting the annotation results and plotting the distribution using R.

### Tandem mass tag (TMT) quantitation

Cells infected with the virus were seeded at a density of 2 × 10^6^ cells per 10 cm dish and incubated overnight. The cells were sent to GENECHEM (Shanghai, China) for TMT quantification. Three independent biological replicates were used for each experiment. Briefly, samples were prepared by adding SDT buffer (4% SDS, 100 mM Tris-HCl, pH 7.6), followed by sonication and heating at 100 °C for 15 min. After centrifugation at 14,000 *g* for 15 min, the supernatant was quantified using a BCA Protein Assay Kit and stored at −80 °C. Proteins (20 µg) were mixed with 6X loading buffer, boiled, and separated by 12% SDS-PAGE. For reduction, 150 µg protein was treated with 100 mM DTT at 100 °C for 5 min, followed by alkylation with iodoacetamide. Proteins were digested overnight with trypsin in NH4HCO3 and the peptides were desalted using a C18 column. Peptides (30 μg) were labeled with TMT and fractionated by RP chromatography. LC-MS/MS analysis was performed on a Q Exactive HF-X mass spectrometer.

### Tumor Xenograft Model

Four-week-old male C-NKG (NOD.Cg-PrkdcscidIl2rgem1cya/Cya, MGI ID:7657569) mice were obtained from Cyagen Biosciences (Suzhou, China). A total (5 × 10^6^ A549 cells were injected subcutaneously into the right dorsal side of mice to establish a tumor model. Mice with A549 allografts were intraperitoneally injected (i. p.) with cisplatin (2 mg/kg), administered every 3 days for a total of four doses. Tumor volume was calculated using the following formula: volume (mm³) = (length × height²)/2. After one month, the mice were euthanized under anesthesia. Subcutaneous tumor tissues were excised, weighed, and sectioned for fluorescent immunohistochemistry (IF-IHC) and immunohistochemical (IHC) staining. Briefly, Tissue sections were deparaffinized in xylene and graded ethanol, followed by antigen retrieval in EDTA buffer (pH 9.0) using microwave heating. Endogenous peroxidase activity was blocked with 3% hydrogen peroxide. The sections were then serum-blocked with 3% BSA and incubated overnight at 4 °C with diluted primary antibody. After washing, an HRP-conjugated secondary antibody was applied and incubated at 37 °C for 20 minutes. Detection was performed using TSA dye (iF-594 Tyramide, Servicebio) or DAB staining, with nuclear counterstaining using DAPI or hematoxylin as appropriate. Finally, the sections were dehydrated using ethanol, cleared in xylene, and mounted with antifade medium or neutral gum for observation under a fluorescence microscope. Antibodies against Cleaved caspase-3 (68773-1-Ig) was obtained from Proteintech. Antibodies against Ki-67 (GB111499) was purchased from Servicebio. Antibodies against phospho-H2AX (Ser139) (2577S, RRID:AB_2118010) was obtained from Cell Signaling Technology. IL-33 (PA5-47006, RRID: AB_2609883) was purchased from Novus and Thermo Fisher Scientific, respectively.

### Tissue microarray

A tissue microarray for lung adenocarcinoma (HLugA180Su12) was obtained from Shanghai OUTDO BIOTECH Co., Ltd., with ethical approval number SHYJS-CP-2206002 provided by the company. The microarray underwent a series of preparations, starting with dewaxing and gradient alcohol rehydration. Antigen retrieval was conducted using sodium citrate at 95 °C for 20 min, followed by natural cooling. After three washes with PBS, the samples were treated with hydrogen peroxide for 20 min, washed again, and then blocked with 5% human serum for 30 min. The primary antibody (5 µg/ml) was applied and incubated at 4 °C overnight. Samples were subsequently returned to room temperature and washed with PBS. They were then incubated with biotin-labeled rabbit anti-goat IgG polymers for 30 min, followed by the application of a streptavidin-biotin peroxidase complex. DAB staining was developed for 35 s and immediately quenched with water, then counterstained with hematoxylin. Finally, the samples underwent gradient alcohol dehydration and were mounted using a neutral resin.

### Statistical analyses

Statistical significance of the *P*-values was assessed using one-way analysis of variance (ANOVA) or an unpaired two-tailed t-test. Statistical significance was set at *P* < 0.05. Survival analysis was performed using the Kaplan-Meier plotter (https://kmplot.com/analysis).

### Ethical approval and consent to participate

Written informed consent was obtained from all participants and the study was approved by the Ethics Committee of the College of Basic Medical Sciences, Jilin University (2024:8).

## Results

### Spatial transcriptomics reveals fibroblasts as the main source of IL-33 in the tumor microenvironment (TME)

**Fig. 1:**
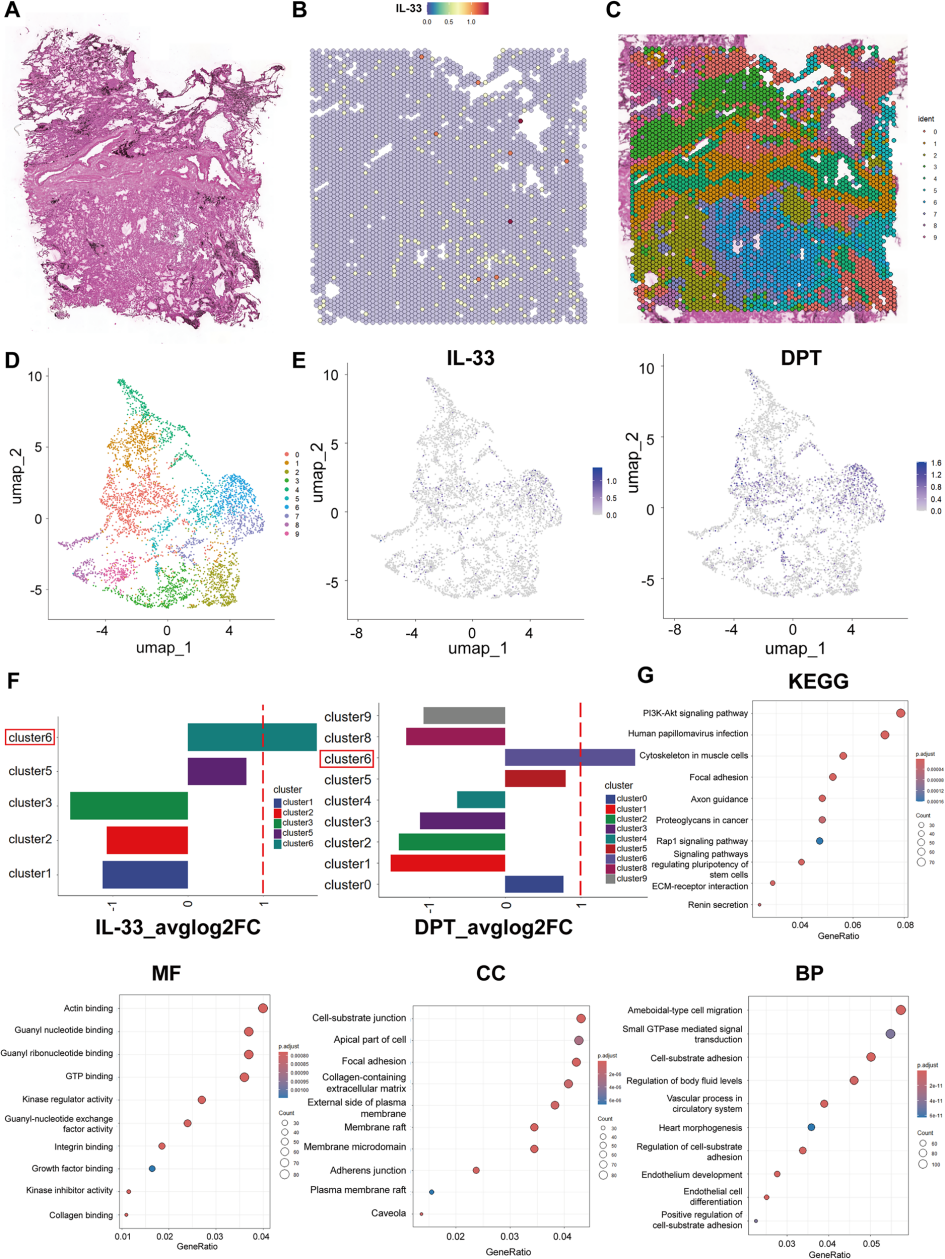
Spatial transcriptomics reveals fibroblasts as the main source of IL-33 in the TME. **A**, **C** H&E staining and UMAP clustering based on spatial transcriptomics of lung adenocarcinoma tumor tissue. **B** Spatial distribution of IL-33 clusters within the tissue. **D** 2D visualization (UMAP dimensionality reduction) of whole-tissue spatial transcriptomics data. **E** UMAP analysis illustrating the distribution and clustering of IL-33 and DPT in lung adenocarcinoma patients based on spatial transcriptomics data. **F** Expression levels of IL-33 and DPT across different clusters, with both highly expressed in cluster 6. **G** Signaling pathways identified through KEGG and GO enrichment analysis comparing cluster 6 to other clusters.

To investigate the distribution of IL-33 in the TME, we utilized the Visium spatial transcriptomics technology from 10X Genomics on biopsy tissue sections from a patient with lung adenocarcinoma (Fig. 1A, B). Transcriptomes from 4511 spots were obtained at a median depth of 3,391 genes per spot (Supplementary Fig. S1). Next, we used UMAP to cluster spots based on global gene expression within individual spots (Fig. 1C, D). As shown in Fig. 1E, F, IL-33 and dermatopontin (DPT) exhibited overlapping expression in cluster 6, with DPT serving as a canonical fibroblast marker. Next, we performed differential gene expression analysis between cluster 6 and other clusters. Kyoto Encyclopedia of Genes and Genomes (KEGG) and Gene Ontology (GO) enrichment analyses further demonstrated the differences between fibroblasts and other cell types in tumor tissues (Fig. 1G). Taken together, these data suggest that IL-33 is primarily expressed in fibroblasts within lung adenocarcinoma tissues.

### CAFs are associated with higher IL-33 expression compared to normal-associated fibroblasts (NAFs)

**Fig. 2:**
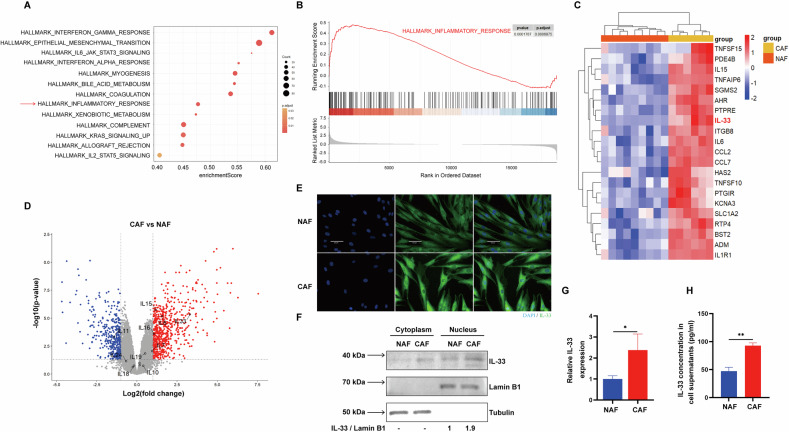
CAFs are associated with higher IL-33 expression compared to NAFs. **A** Pathway analyses for CAFs versus NAFs primarily utilized HALLMARK gene sets from the Molecular Signatures Database (MSigDB). **B** GSEA analysis of RNA-seq data showing enrichment of inflammatory response genes signature in CAFs. **C** Heat map analysis of inflammatory response genes in CAFs and NAFs. **D** Volcano plot depicting the gene expression profile of CAFs and NAFs, with differentially expressed genes labeled in red (fold change >2) and blue (fold change <−2). **E** Immunofluorescence (IF) staining of IL-33 in CAFs and NAFs. Representative images show anti-IL-33 antibody (green) and DAPI counterstaining (blue). Scale bars represent 60 μm. **F** Cell lysates from NAFs and CAFs were separated using a nucleus-cytoplasm separation kit and subjected to immunoblotting (IB), with Lamin B1 and alpha-tubulin as markers for nuclear and cytoplasmic fractions, respectively. **G** NAFs and CAFs were harvested for qRT-PCR. **H** Cell culture supernatants from NAFs and CAFs were collected for ELISA. Data were shown as mean ± SD and were analyzed by one-way ANOVA or unpaired two-tailed t-test. * *P* < 0.05, ** *P* < 0.01, *** *P* < 0.001.

Extensive evidence has indicated that CAFs can promote tumor growth, invasion, metastasis, and drug resistance through various pathways. To explore the differences between NAFs and CAFs, we compared their expression profiles using publicly available data [31]. Using hallmark enrichment analysis, we found that the inflammatory gene signature was highly enriched in CAFs compared to NAFs (Fig. 2A). Gene Set Enrichment Analysis (GSEA) showed significant positive enrichment for the inflammatory response in CAFs, and heat map analysis revealed that these genes were markedly upregulated in CAFs compared to NAFs (Fig. 2B, C). Notably, IL-33 is a cytokine that exhibits notable changes (Fig. 2C, D). To further confirm these findings, we observed that the expression of IL-33 in CAFs was significantly higher than that in NAFs, both in the cytoplasm and nucleus (Fig. 2E, F and Supplementary Fig. S2A). Moreover, CAFs exhibited not only elevated IL-33 protein expression but also higher mRNA levels, along with increased IL-33 protein secretion into the culture supernatant (Fig. 2G, H). Collectively, we demonstrated that CAFs exhibit increased expression and secretion of IL-33 compared with NAFs.

### IL-33 in the TME promotes chemoresistance of tumor cells

**Fig. 3:**
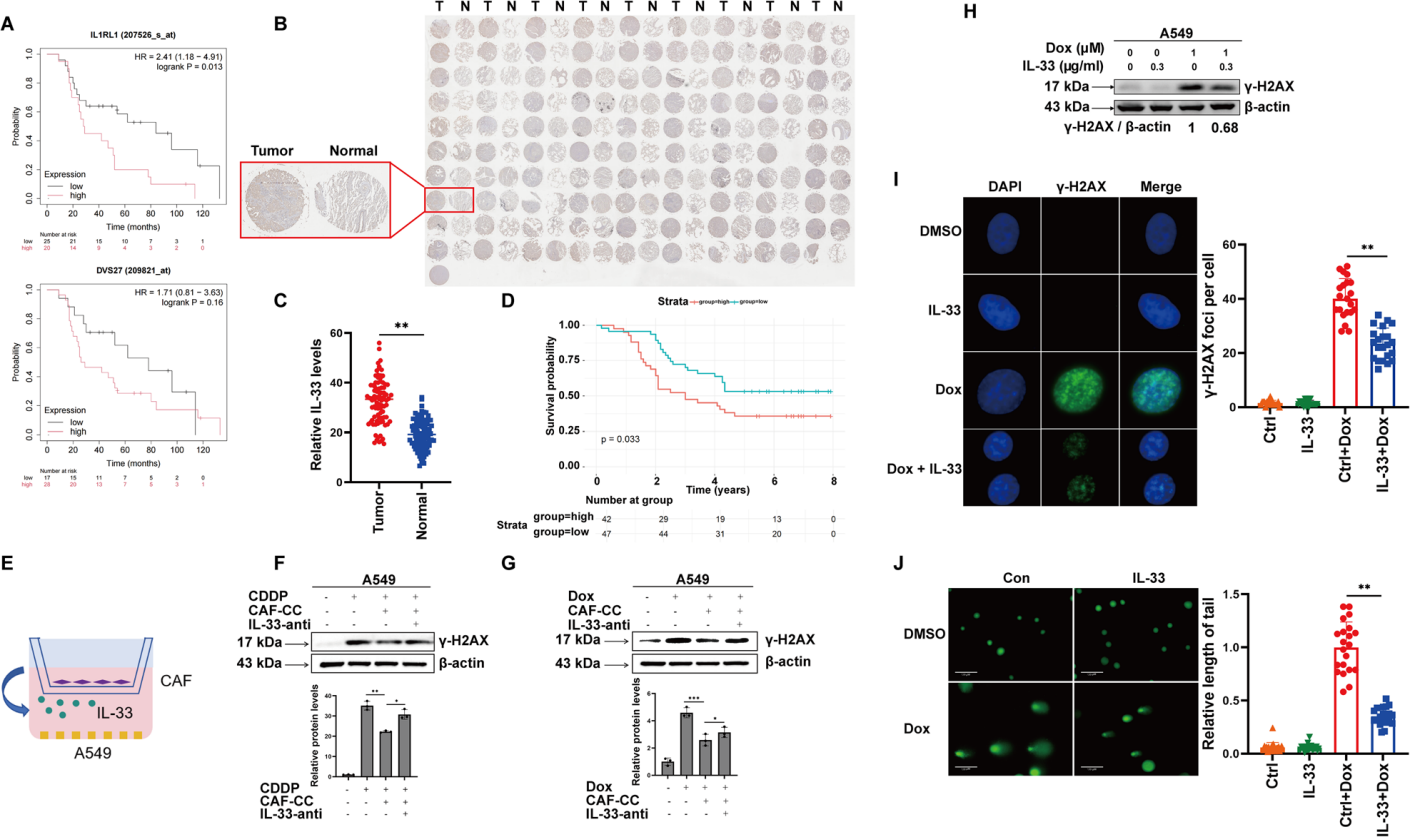
IL-33 in the TME promotes chemoresistance of tumor cells. **A** Kaplan-Meier survival curve illustrating overall survival (OS) data from lung cancer patients. **B** The comparison of IL-33 protein expression between normal and cancerous tissues was conducted using IHC staining on a tissue microarray composed of 90 lung adenocarcinoma samples along with normal lung tissues. **C** Quantitative analysis of IL-33 protein expression levels in tissue microarrays was conducted using ImageJ. **D** A Kaplan−Meier survival curve was generated to illustrate the OS data of lung cancer patients represented in the tissue microarray. **E** Schematic diagram depicting the co-culture experiments. **F** A549 cells, either in single culture or co-cultured with CAFs, were treated with 3 μM CDDP or in combination with IL-33 neutralizing antibody for 48 h, and then cells were harvested for IB analysis. (**G**) A549 cells, either in single culture or co-cultured with CAFs, were treated with 1 μM Dox or in combination with IL-33 neutralizing antibody for 48 h, and then cells were harvested for IB analysis. **H** A549 cells were treated with 1 μM Dox or in combination with IL-33 recombinant protein for 48 h, and then cells were harvested for IB analysis. **I** A549 cells were treated with 1 μM Dox or in combination with IL-33 recombinant protein for 48 h and then cells were harvested for IF staining. **J** A549 cells were treated with 1 μM Dox or in combination with IL-33 protein for 48 h, and then cells were harvested for alkaline comet assay.

We used the Kaplan-Meier Plotter website to explore the clinical relevance of IL-33 expression in lung adenocarcinoma (LUAD). Interestingly, higher expression levels of *IL1RL1* (gene encoding ST2) and *IL-33(DVS27)* correlated with shorter overall survival in patients who received radiotherapy and chemotherapy, suggesting that IL-33 may mediate the DNA damage response (DDR) of tumor cells (Fig. 3A). To further validate these results, we performed a tissue microarray containing lung adenocarcinoma samples from 89 patients with complete survival data. Our immunohistochemical analysis revealed that the protein expression levels of IL-33 are significantly higher in cancers than those in adjacent non-cancerous tissues (Fig. 3B, C) and higher IL-33 expression in tumors significantly correlates with shorter five- year survival of patients (Fig. 3D). Since the results above indicate that IL-33 primarily originates from CAFs in the TME (Fig. 1), we employed a non-contact (Transwell) co-culture of CAFs and A549 cells (Fig. 3E). Cells were treated with cisplatin (CDDP) or doxorubicin (DOX) for 48 h to induce extensive DNA damage. We found that A549 cells co-cultured with CAFs exhibited dramatically reduced sensitivity to drug-induced DNA damage, an effect that could be reversed by the addition of IL-33 neutralizing antibody, as indicated by γ-H2AX levels (Fig. 3F, G). To further validate whether IL-33 promotes DDR, tumor cells were treated with recombinant IL-33. Recombinant IL-33 treatment significantly enhanced DDR, as illustrated by lower expression levels and fewer foci of γ-H2AX compared to the IL-33-deficient group (Fig. 3H, I). In addition, we performed an alkaline comet assay to more accurately measure the level of DNA damage. Consistently, cells treated with recombinant IL-33 protein exhibited shorter tail moments, indicating less DNA damage after Dox treatment (Fig. 3J). Overall, these data suggest that IL-33 in the TME promotes chemoresistance in tumor cells by mediating DDR.

### IL-33 overexpression reduces the efficacy of chemotherapeutic drugs in tumor cells

**Fig. 4:**
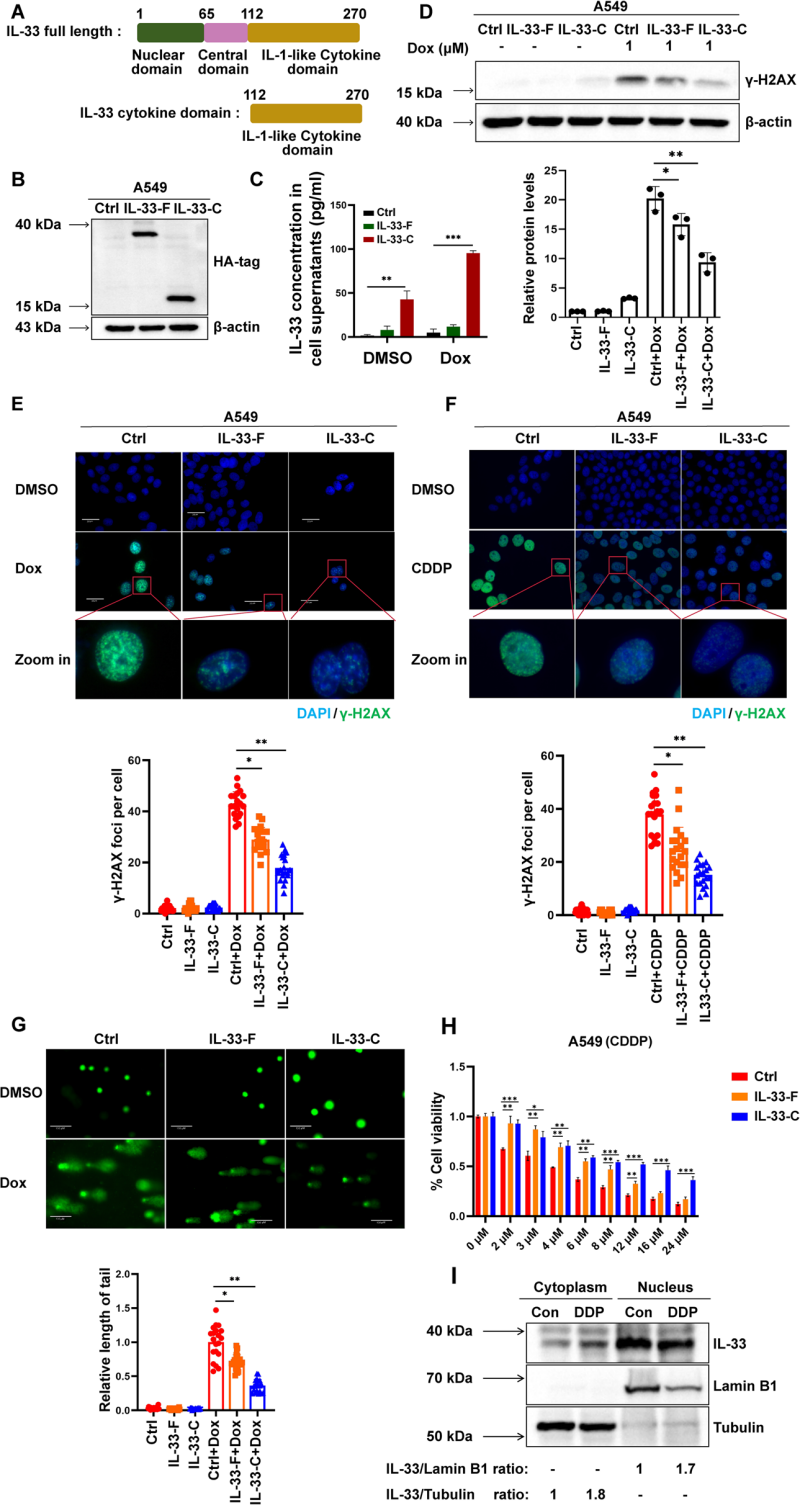
IL-33 overexpression reduces the efficacy of chemotherapeutic drugs in tumor cells. **A** Schematic representation of IL-33 full-length and IL-33 cytokine domain constructs. **B** IB analysis was performed using virus-infected A549 cells, including control (Ctrl), IL-33-F OE, and IL-33-C OE groups. **C** Detection of IL-33 in the cell culture supernatants of A549 cells (Ctrl, IL-33-F OE, and IL-33-C OE) was performed using ELISA. **D** Virus-infected A549 cells (Ctrl, IL-33-F OE, and IL-33-C OE) were treated with or without 1 μM Dox for 48 h, and then cells were harvested for IB analysis and quantification of IB band density using ImageJ. **E** Virus-infected A549 cells (Ctrl, IL-33-F OE, and IL-33-C OE) were treated with or without 1 μM Dox for 48 h, and then cells were harvested for IF staining. **F** Virus-infected A549 cells (Ctrl, IL-33-F OE, and IL-33-C OE) were treated with or without 3 μM CDDP for 48 h, and then cells were harvested for IF staining. **G** Virus-infected A549 cells (Ctrl, IL-33-F OE, and IL-33-C OE) were treated with or without 1 μM Dox for 48 h, and then cells were harvested for alkaline comet assay. **H** Virus-infected A549 cells (Ctrl, IL-33-F OE, and IL-33-C OE) cells were treated with different concentrations of CDDP as indicated for 72 h, and the viability of cells was determined by the MTT assay. **I** Cell lysates from Control A549 cells and DDP were separated using a nucleus-cytoplasm separation kit and subjected to IB.

Recent studies have indicated that lung cancer cells can release IL-33, promote M2 macrophage polarization, and enhance tumor cell immune escape [32]. Next, we evaluated whether IL-33 from tumor cells could affect the efficacy of chemotherapy. First, we generated IL-33 full-length (IL-33-F) and IL-33 cytokine domain (IL-33-C) constructs and expressed them in A549 cells (Fig. 4A, B). Consistent with previous studies, IL-33-F was localized predominantly in the nucleus, while IL-33-C resided in both the nucleus and cytoplasm (Supplementary Fig. S2B). Additionally, ELISA experiments demonstrated that overexpression (OE) of IL-33-C in A549 cells resulted in greater IL-33 release than that in the other groups. Notably, Dox treatment further increased IL-33 levels in the supernatant (Fig. 4C). Importantly, we found that OE of either IL-33-F or IL-33-C in A549 cells attenuated DOX-induced DNA damage, as illustrated by fewer foci and lower expression levels of γ-H2AX compared to those in the control group (Fig. 4D, E). Similar results were observed in another lung adenocarcinoma cell line, H1299, as well as CDDP-induced DNA damage model (Supplementary Fig. S2E, S2F and Fig. 4F). To further confirm these data, we examined the DNA damage levels and cell proliferative potential following these treatments using comet and colony formation assays, respectively. As shown in Fig. 4G and Supplementary Fig. S2C, both IL-33-F and IL-33-C OE reduced DNA damage and enhanced in vitro cellular growth following chemotherapeutic drug treatment. This finding was further supported by the cell viability assays and Annexin V/PI staining (Fig. 4H, Supplementary Fig. S2D and S2H). Of note, conditioned medium (CM) collected from the supernatant of IL33-C OE A549 cells, could also enhance the DDR of parental A549 cells as demonstrated by the reduced levels of γH2AX (Supplementary Fig. S2G). Moreover, we established A549 cisplatin-resistant cell lines (DDP) to explore changes of IL-33 expression in these resistant cell lines (Supplementary Fig. S2I-S2K). Interestingly, we observed that IL-33 expression in DDP cells was significantly higher than that in parental cells, both in the cytoplasm and nucleus (Fig. 4I). Taken together, these data indicate that IL-33 expression in lung cancer cells is negatively correlated with the efficacy of DNA damage-inducing chemotherapeutic drugs.

### IL-33 drives resistance to chemotherapeutic drugs through activation of the MAPK/NHEJ pathway

**Fig. 5:**
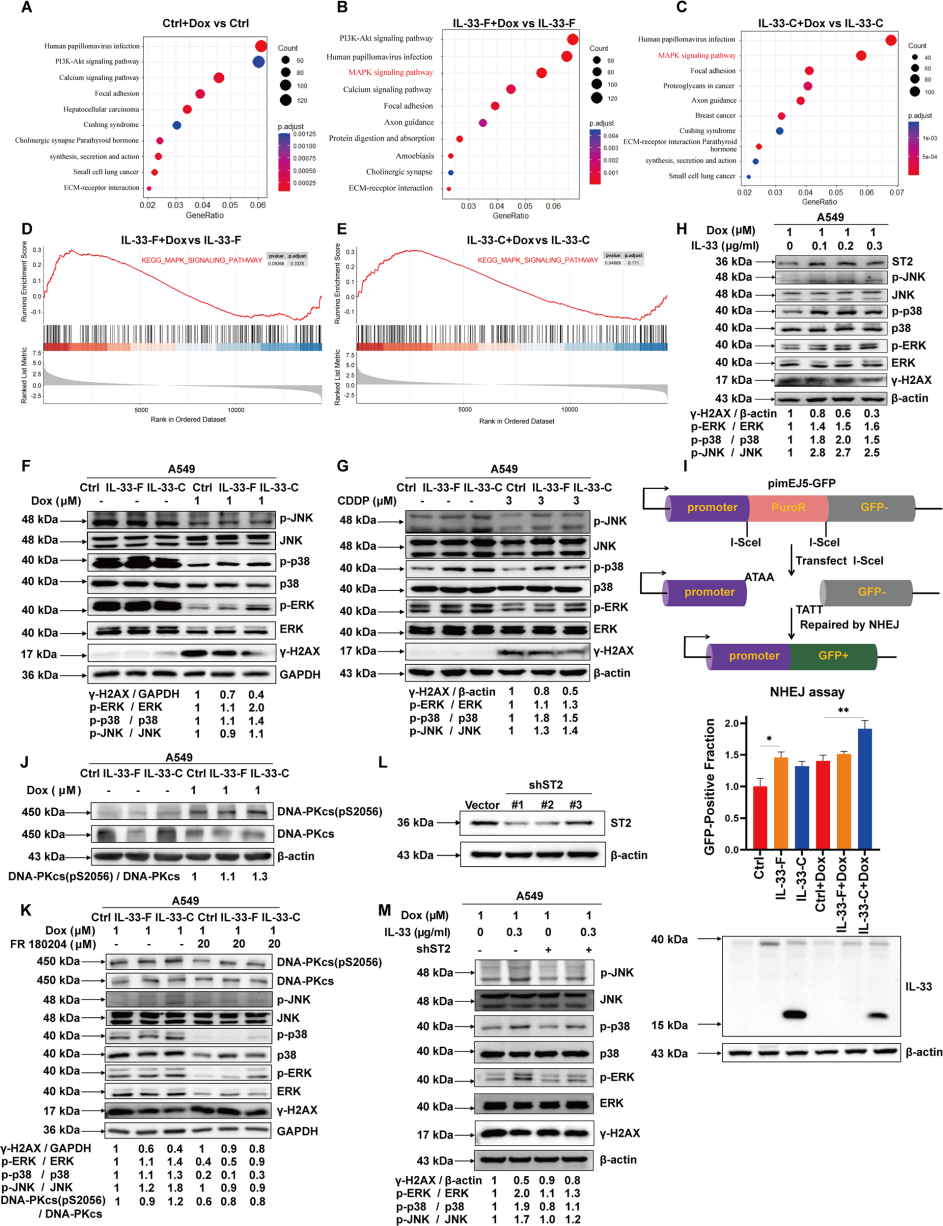
IL-33 drives resistance to chemotherapeutic drugs through activation of the MAPK/NHEJ pathway. **A**, **B**, **C** Signaling pathways identified through KEGG enrichment analysis comparing A549 cells (Ctrl, IL-33-F OE, and IL-33-C OE) treated with and without Dox. **D** GSEA analysis of RNA-seq data showing enrichment of the MAPK gene signature in A549 IL-33-F OE treated with Dox. **E** GSEA analysis of RNA-seq data showing enrichment of the MAPK gene signature in A549 IL-33-C OE treated with Dox. **F** Virus-infected A549 cells (Ctrl, IL-33-F OE, and IL-33-C OE) were treated with or without 1 μM Dox for 48 h, and then cells were harvested for IB analysis. **G** Virus-infected A549 cells (Ctrl, IL-33-F OE, and IL-33-C OE) were treated with or without 3 μM CDDP for 48 h, and then cells were harvested for IB analysis.**H** A549 cells were treated with 1 μM Dox in combination with different concentrations of IL-33 recombinant protein for 48 h, and then harvested for IB analysis. **I** Upper panel, Schematic of the pimEJ5-GFP vector-based assay. Lower panels, A549 cells were transfected with pimEJ5-GFP and HA-Sce1. The GFP-positive fraction of cells indicates the frequency of NHEJ repair, while IB analysis was performed simultaneously to confirm the expression of IL-33-F OE and IL-33-C OE. For flow cytometry experiments, at least three independent assays were conducted. **J** Virus-infected A549 cells (Ctrl, IL-33-F OE, and IL-33-C OE) were treated with or without 1 μM Dox for 48 h, and then cells were harvested for IB analysis. **K** A549 cells were treated with 1 μM Dox in combination with or without 20 μM MAPK inhibitor FR180204 for 48 h, and then harvested for IB analysis. **L** Virus-infected A549 cells (Vector, shST2 #1, shST2 #2, and shST2 #3) were harvested for IB analysis. **M** Virus-infected A549 cells (Vector and shST2 #1) were treated with 1 μM Dox or in combination with IL-33 recombinant protein for 48 h, and then cells were harvested for IB analysis.

To explore the potential mechanism by which IL-33 contributes to DDR, we performed RNA sequencing experiments in cells expressing IL-33-F or IL-33-C OE, as well as their respective samples after Dox treatment. KEGG enrichment analysis indicated that the MAPK signaling pathway was among the top three most significantly enriched pathways after Dox treatment in both IL-33-F and the IL-33-C overexpressing, but not in vector-infected A549 cells (Fig. 5A−C). Consistently, GSEA using the ‘MAPK signaling pathway’ signature genes further demonstrated that both IL-33-F and IL-33-C expression levels were positively associated with the MAPK pathway in doxorubicin-treated tumor cells (Fig. 5D, E). We then validated the bioinformatic data using western blot analysis. The results indicated that IL-33 OE increased the expression levels of the phosphorylated forms of ERK, p38, and JNK, with the IL-33 C-OE group showing the most obvious effect. (Fig. 5F, G). Furthermore, we found that recombinant IL-33 protein treatment reduced DOX-induced DNA damage and enhanced activity of the MAPK pathway in a dose-dependent manner (Fig. 5H). Interestingly, IL-33 seemed creating a positive feedback loop on its own as exogenous IL-33 treatment increased both the protein expression levels of IL-33 and ST2 in A549 cells (Fig. 5H and Supplementary Fig. S2L). Since previous studies have reported that ERK phosphorylation is correlated with DNA damage repair [33], with ERK activation involved in promoting the phosphorylation of DNA-PK (cs) and activating non-homologous end joining (NHEJ) repair, we next examined the role of IL-33 in regulating the NHEJ pathway. IL-33-C OE was associated with increased expression of phosphorylated forms of DNA-PK (cs) and enhanced cellular NHEJ repair activity, as evidenced by the pimEJ5GFP plasmid-based assay and western blot analysis (Fig. 5I, J). Moreover, we found that treatment with MAPK inhibitors (FR 180204 and SB 203580) partially reversed the enhanced DNA damage repair efficiency induced by IL-33 OE (Supplementary Fig. S3A and Fig. 5K). Furthermore, we performed ST2 silencing experiments to confirm that ST2 deletion reduced the effect of IL-33 in stimulating MAPK activation and DNA damage repair (Fig. 5L, M). In summary, the data presented above suggest that IL-33 stimulates the MAPK pathway, which can subsequently lead to activation of the NHEJ pathway in tumor cells and confer resistance to chemotherapeutic drugs.

### Nuclear IL-33 is involved in DNA double strand breaks (DSBs) repair

**Fig. 6:**
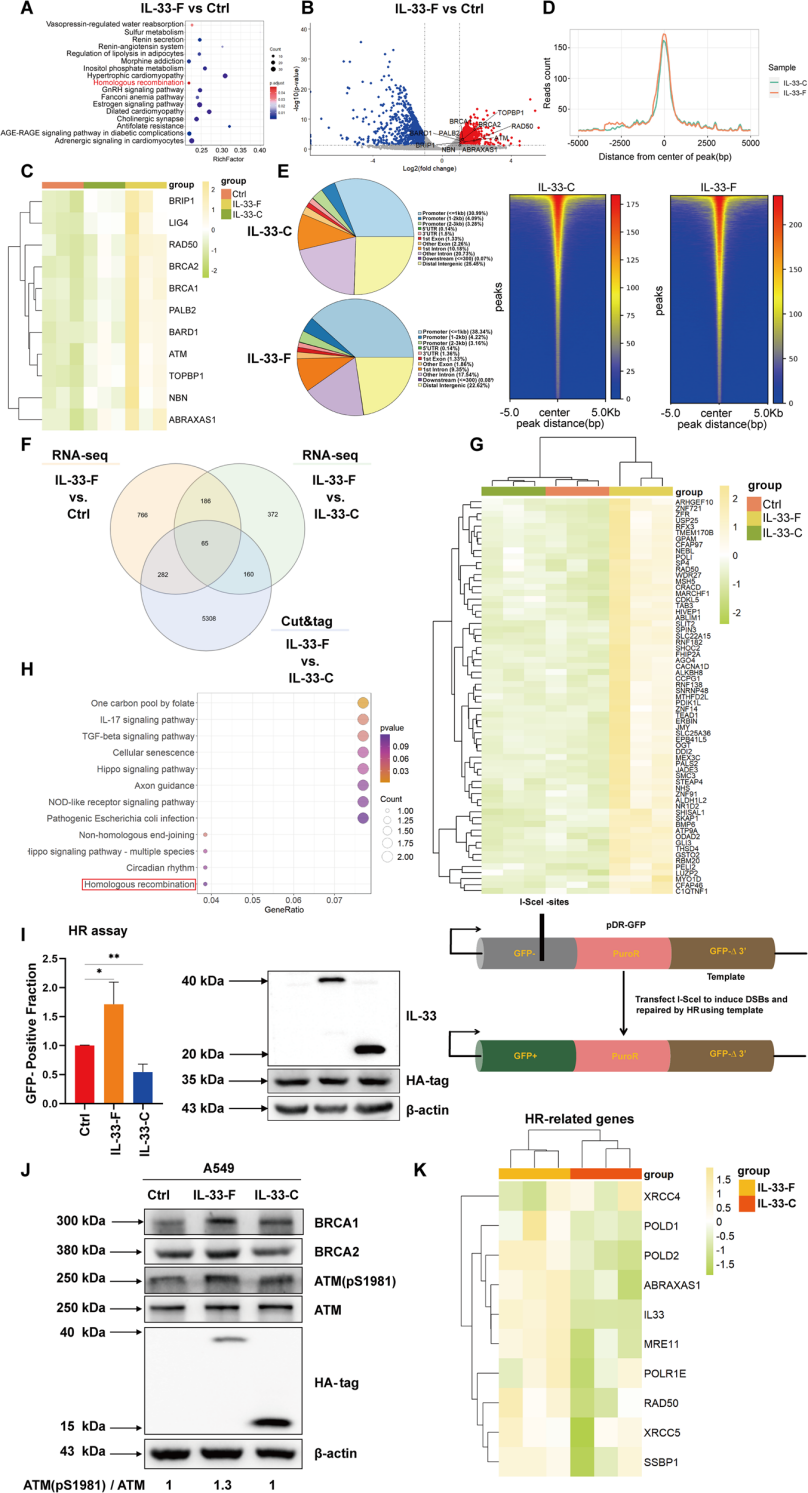
Nuclear IL-33 is involved in DNA double strand breaks (DSBs) repair. **A** Enrichment of KEGG signatures for IL-33-F OE regulated genes. **B** Volcano plot depicting the gene expression profile of IL-33-F OE and Ctrl, with differentially expressed genes labeled in red (fold change >2) and blue (fold change <−2). **C** Heat map analysis of HR hallmark genes in A549 cells. **D** Averaged intensities (upper panel) and centered read density heatmaps (lower panel) of CUT&Tag signals for the binding of IL-33-F (HA tag) and IL-33-C (HA tag). **E** A pie chart displaying genomic annotation of peaks in A549 cells for IL-33-C OE (top) and IL-33-F OE (bottom). **F** Venn diagram illustrating the differential genes shared between RNA-Seq and CUT&Tag results. For the CUT&Tag analysis, A549 cells infected with IL-33-F were compared to those infected with IL-33-C. **G** Heat map analysis of 65 overlapping genes in the A549 cell model. **H** Enrichment of KEGG signatures for overlapping gene. **I** Schematic of the pDR-GFP vector-based assay (right). A549 cells were transfected with pDR-GFP and HA-Sce1. The GFP-positive fraction of cells indicates the frequency of HR repair, while IB analysis was performed simultaneously to confirm the expression of IL-33-F OE, IL-33-C OE, and Sce1. For flow cytometry experiments, at least three independent assays were conducted (left). **J** Virus-infected A549 cells (Ctrl, IL-33-F OE, and IL-33-C OE) were harvested for IB analysis. **K** Heat map analysis of protein levels of HR-related genes in A549 cells using TMT quantitation data.

Given that IL-33-F is primarily localized in the nucleus and several studies have indicated that nuclear IL-33 can regulate gene transcription [34**–**36], we performed RNA sequencing experiments to explore whether IL-33-F could regulate DDR through a mechanism different from the release of bioactive IL-33. Remarkably, KEGG gene set analysis revealed that compared with control cells, the homologous recombination (HR) pathway was among the top ten most significantly enriched pathways in IL-33-F OE A549 cells (Fig. 6A). Next, we employed volcano plot and heat map analysis using the ‘homologous recombination’ signature genes and observed that IL-33-F expression was positively correlated with the HR pathway in A549 cells (Fig. 6B, C). qPCR analysis of core HR genes exemplified by BRCA1 and BRCA2 further confirmed this observation (Supplementary Fig. S3G). In addition, we performed a cleavage under targets and mentation (CUT&Tag) assay to further examine the nuclear function of IL-33. IL-33-F showed greater enrichment at the promoters of genes than IL-33-C did (Fig. 6D, E). By cross-comparing the RNA-Seq and CUT&Tag data, we narrowed down the nuclear IL-33 candidate targets to 65 genes (Fig. 6F). As shown in Fig. 6G, these candidate genes were significantly upregulated following IL-33-F OE but not IL-33-C OE in A549 cells. Notably, the upregulated genes were highly enriched in the HR pathway (Fig. 6H). Consistent with the bioinformatic data presented above, the IL-33-F OE group exhibited enhanced HR repair activity compared to the other groups, as demonstrated by the cellular HR assay (Fig. 6I). Moreover, IL-33-F OE significantly increased the expression levels of crucial HR factors, as exemplified by BRCA1 and BRCA2, as well as the phosphorylation level of ATM, which serves as a marker for enhanced HR repair activity (Fig. 6J). Using whole-cell tandem mass tag (TMT) quantitation, we found that IL-33-F OE significantly increased the protein levels of several HR-related factors, including POLD1, POLD2, RAD50, ABRAXAS1, XRCC5, XRCC4, MRE11, POLR1E, and SSBP1, compared to those in the IL-33-C OE group (Fig. 6K). Consistently, IL-33-F bound to open chromatin areas of HR-related genes with higher efficiency than IL-33-C, considering the difference in their basal expression levels during the CUT & Tag assay. (Supplementary Fig. S3B and S3C). Using data from TMT, GO analysis indicated that the DNA repair complex pathway was among the top three most significantly enriched pathways in IL-33-F OE A549 cells compared to IL-33-C OE cells (Supplementary Fig. S3D). Furthermore, GSEA and heatmap analysis showed that IL-33-F expression was positively associated with the DNA repair capacity of A549 cells (Supplementary Fig. S3E and S3F). Altogether, these data suggest that nuclear IL-33 can promote DSBs repair through the regulation of HR repair, in addition to its role in enhancing the levels of the bioactive form of IL-33.

### IL-33 promotes lung cancer resistance to CDDP in vivo

**Fig. 7:**
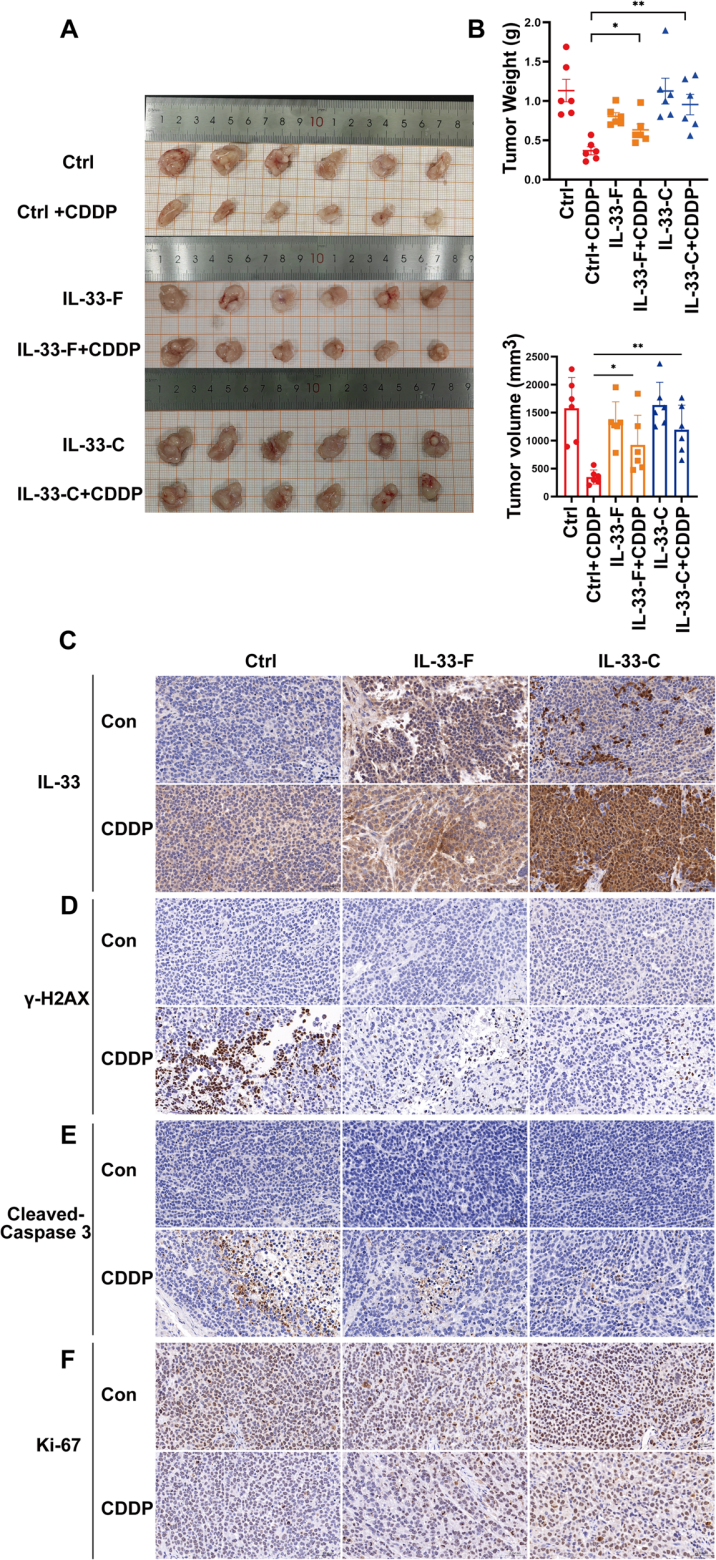
IL-33 promotes lung cancer resistance to CDDP in vivo. Images of tumors obtained from C-NKG mice. **B** Upper panel, tumor weights of C-NKG mice. Data points represent the average tumor weight (g) in each group (*n* = 6). Lower panel, tumor volumes obtained from C-NKG mice. Data points represent the average tumor volume (mm³) in each group (*n* = 6). **C** IL-33 IHC staining of tumor sections from each group. **D** γ-H2AX IHC staining of tumor sections from each group. **E** Cleaved-Caspase 3 IHC staining of tumor sections from each group. **F** Ki-67 IHC staining of tumor sections from each group. Scale bars represent 50 μm.

To assess the role of IL-33 in the regulation of chemoresistance in vivo, we conducted a xenograft study using A549 cells stably infected with IL-33-F or IL-33-C. As shown in Fig. 7A, B, overexpression of IL-33-F and IL-33-C significantly reduced the antitumor effect of CDDP in A549 xenograft models compared to that in the control group, with IL-33-C OE showing the most obvious effect. Moreover, consistent with our in vitro data, fluorescent immunohistochemistry (IF-IHC) and immunohistochemistry (IHC) staining revealed that cisplatin treatment significantly increased IL-33 release in tumor tissues (Fig. 7C and Supplementary Fig. S4A). In addition, overexpression of IL-33-F and IL-33-C significantly downregulated the protein expression of γ-H2AX and cleaved caspase-3 but increased Ki-67 in tumor tissues, suggesting that IL-33 ameliorated DNA damage, apoptosis, and growth inhibition induced by CDDP treatment in vivo (Fig. 7D−F and Supplementary Fig. S4B−S4D).

## Discussion

In this study, we demonstrated that IL-33 within the tumor microenvironment (TME), primarily secreted by cancer-associated fibroblasts (CAFs), can enhance DNA damage-resistance in tumor cells by activating the MAPK/NHEJ pathway. Exposure to DNA-damaging agents leads to increased IL-33 expression in tumor cells. Elevated IL-33 expression is associated with reduced efficacy of chemotherapeutic drugs in tumor cells, as nuclear IL-33 is also implicated in the repair of DNA double-strand breaks. Additionally, IL-33 from the TME could further upregulate IL-33 and ST2 expression within the tumor cells themselves, forming its own positive feedback regulation (Fig. 5H and Supplementary Fig. S2L). Coculturing with CAFs significantly increased the expression levels of IL-33 in A549 cells under normal or drug-treated conditions (Supplementary Fig. S5A). Of note, after DNA-damaging agent treatment, the protein expression level of IL-33 in CAFs was sharply decreased, which is consistent with the observation that more IL-33 was secreted, subsequently, stimulate the A549 cells, further increased the amount of IL-33 in the supernatant. (Supplementary Fig. S5B and S5C). Our findings suggest that targeting IL-33 mediated pathway may be a promising approach to mitigate resistance to chemotherapy and radiotherapy.

IL-33 is well known for its role in regulating type 2 innate immune responses and inflammation by interacting with immune cells such as regulatory T cells (Tregs), macrophages, and group 2 innate lymphoid cells (ILC2s). However, given the complexity of the TME, IL-33 appears to play a controversial role during cancer progression in a context-dependent manner. In this study, we demonstrated that both CAFs and tumor cells treated with DNA damage-inducing agents secrete high levels of IL-33, which promotes DDR and chemoresistance in lung cancer cells (Supplementary Fig. S5B, S5C and Fig. 4C). Notably, CAF-CM-induced chemoresistance was largely reversed by adding IL-33 neutralizing antibodies. Moreover, in LUAD patients, higher expression levels of *IL1RL1* and *IL-33* were correlated with shorter overall survival in patients who received chemotherapy and radiotherapy.

Recently, IL-33 has been reported to be actively involved in promoting the therapeutic resistance of cancer cells by several groups. First, cancer stem cells can establish an IL-33-TGF-β niche signaling loop to drive immunosuppressive TME and drug resistance [37, 38]. Consistently, IL-33 has been shown to promote stemness and prevent chemotherapy-induced tumor apoptosis in colon cancer, myeloid leukemia, and glioma models [39**–**41]. In addition, Yang et al. reported that the IL-33/NF-κB/ST2L/Rab37 positive-feedback loop promotes M2 macrophage polarization to limit chemotherapeutic efficacy in lung cancer [32]. Interestingly, Tregs accumulation in the TME can also be mediated by the IL-33/ST2 axis, further indicating this IL-33/ST2 axis may be a potential target for enhancing cancer therapy [42]. Herein, we show that IL-33/ST2 stimulates the NHEJ pathway and resistance to DNA damage-inducing chemotherapeutic drugs in tumor cells, providing a direct link between abnormal DDR and the IL-33-mediated network.

Notably, the findings listed above have mainly focused on the IL-33/ST2 axis or the secreted form of IL-33. Compared to the canonical role of the IL-33/ST2 axis, the nuclear function of IL-33 is much less understood. IL-33-F harbors a nuclear localization signal and a homeodomain that can bind to heterochromatin, which is a prerequisite for its potential transcriptional regulatory role [34]. Remarkably, Park et al. identified that nuclear IL-33 repressed *Smad6* gene expression, which led to enhanced TGF-β/SMAD signaling pathway activation and tumor development [36]. To our knowledge, a previous study has also shown that IL-33 can act as a transcriptional repressor, but the transcriptional activating role of IL-33 has not yet been reported [35]. To better decipher the role of IL-33 in transcriptional regulation, we performed an integrated analysis using Cut & Tag, RNA-seq, and TMT quantitation. Surprisingly, we found that the HR pathway was among the top ten most significantly enriched pathways after IL-33-F OE. Consistently, crucial HR factors and cellular HR activity were upregulated after IL-33-F treatment. Considering the fundamental role of genomic instability adaptation during the establishment of therapeutic resistance, our work may provide much-needed insight into the transcriptional regulation of abnormal DDR in the context of chronic inflammation and/or an immunosuppressive TME. Notably, we noticed that IL-33-C also displayed certain levels of nuclear expression and chromatin binding (Supplementary Fig. S2B and Fig. S3C). Unfortunately, we could not further determine the mechanism of nuclear residence of IL-33-C and its potential transcriptional regulatory role in this study.

Interestingly, we observed enhanced expression of IL-33 in A549 cisplatin-resistant cell lines, and cells with IL-33-F OE also released increased levels of shortened IL-33 in the supernatant (Fig. 4C). Thus, we speculate that the increased expression of IL-33 in tumor cells may contribute to abnormal DDR through transcriptional regulation of HR genes and IL-33/ST2/MAPK-mediated NHEJ activation. However, we were not able to further explore the mechanisms by which the choice between these two crucial DSBs repair pathways is determined after high IL-33-F expression, although previous studies have indicated that cell cycle control and phosphorylation of repair proteins are crucial for regulating this balance [43]. According to our in vitro and in-vivo results, IL33-C OE cells exhibited stronger resistance to chemotherapeutic drugs than IL33-F OE cells. We propose that cells with IL-33-C OE can release much higher concentrations of bioactive IL-33 upon drug treatment, which may play a dominant role in the DDR. While we have demonstrated that IL-33-F can drive the HR activity without treating with DNA-damaging agents, increased activity of NHEJ repair under bioactive IL-33 stimulation might be more important during the development of chemoresistant TME, especially considering the role of NHEJ in driving genomic instability. Nevertheless, due to the limitations of this study, the fine-tuned regulation of IL-33 release after chemotherapy and its downstream network requires further investigation. In brief, although the exact mechanism of enhanced IL-33 expression remains undetermined, we were able to show that hyperactivation of IL-33 facilitates a DNA damage-resistant TME through both the paracrine pathway from CAFs and the autocrine pathway from tumor cells (Supplementary Fig. S6 model).

In summary, our work demonstrated that IL-33 plays a key role in mediating a DNA damage-resistant TME, which could represent a potential therapeutic vulnerability in targeting chemoresistant cancer cells.

## Supplementary information

Table S1

Supplementary figure legends

Supplemental figures

patients' data of tissue microarray

Full western blots images

## Data Availability

Genomic datasets of this study, including those of RNA-sequencing and CUT&Tag, have been deposited in the NCBI Gene Expression Omnibus (GEO) database under the accession number GSE282699. The spatial transcriptomics of lung cancer patient data reported in this paper have been deposited in the Genome Sequence Archive [29] in National Genomics Data Center [30], China National Center for Bioinformation / Beijing Institute of Genomics, Chinese Academy of Sciences (GSA-Human: HRA009465) that are publicly accessible at https://ngdc.cncb.ac.cn/gsa-human. The TMT data reported in this paper have been deposited in the OMIX, China National Center for Bioinformation / Beijing Institute of Genomics, Chinese Academy of Sciences (https://ngdc.cncb.ac.cn/omix: accession no. OMIX008019. The Publicly available data generated by other researchers were used in this study, including RNA-seq data (GSE164750), A549 ATAC-seq data (GSE252848), and A549 ChIP-seq data (GSE186186), all of which were obtained from the NCBI GEO database. All data, codes, and materials are available from the corresponding author upon request.
