# Supplementary material for: Interleukin-33 (IL-33) promotes DNA damage-resistance in lung cancer
Source: Cell Death Dis. 2025 Apr 11;16(1):274. doi: 10.1038/s41419-025-07624-x (PMC11992107; doi:10.1038/s41419-025-07624-x)
Supplement: Supplementary file 1 — Table S1 [file 41419_2025_7624_MOESM1_ESM.pdf]

Table S1. The sequences of quantitative real-time PCR primers.

Gene	Forward primer	Reverse primer
IL33	GCCTTGTGTTTCAAGCTGGG	CCAAAGGCAAAGCACTCCAC
BRCA1	GAAACCGTGCCAAAAGACTTC	CCAAGGTTAGAGAGTTGGACAC
BRCA2	CACCCACCCTTAGTTCTACTGT	CCAATGTGGTCTTTGCAGCTAT
β-actin	CCTGGCACCCAGCACAAT	GGGCCGGACTCGTCATAC
