# Supplementary material for: Interleukin-33 (IL-33) promotes DNA damage-resistance in lung cancer
Source: Cell Death Dis. 2025 Apr 11;16(1):274. doi: 10.1038/s41419-025-07624-x (PMC11992107; doi:10.1038/s41419-025-07624-x)
Supplement: Supplementary file 2 — Supplementary figure legends [file 41419_2025_7624_MOESM2_ESM.docx]

**Supplementary figure legends.**

**Supplementary Figure 1**

Summary of spatial transcriptomics.

**Supplementary Figure 2**

(A) Morphological features of primary fibroblasts isolated from lung tissue and lung cancer tissue. Scale bar: 100 μm. (B) Representative images showing the cellular localization of IL-33 expression constructs, with anti-HA antibody marking the IL-33 constructs. (C) Colony formation assays were conducted in virus-infected A549 cells (Ctrl, IL-33-F OE, and IL-33-C OE) exposed to various concentrations of CDDP for two weeks. (D) Virus-infected A549 cells (Ctrl, IL-33-F OE, and IL-33-C OE) were treated with different concentrations of Dox for 72 hours, and cell viability was determined by the MTT assay. (E) IB analysis was performed using virus-infected H1299 cells, including Ctrl, IL-33-F OE, and IL-33-C OE groups. (F) Virus-infected H1299 cells (Ctrl, IL-33-F OE, and IL-33-C OE) were treated with 5 μM Dox for 48h, and then cells were harvested for IB analysis. (G) A549 cells were treated with 1 μM Dox or in combination with the conditioned medium from virus-infected A549 cells (IL-33-C OE) for 48 hours, and then cells were harvested for IB analysis. (H) Virus-infected A549 cells (Ctrl, IL-33-F OE, and IL-33-C OE) were treated with or without 1 μM Dox for 48 hours, then harvested for Annexin V/PI staining. Annexin V-positive cells were quantified as apoptotic cells. At least three independent experiments were performed for each sample. (I) A549 and DDP cells were treated with or without 3 μM CDDP for 72 hours, and cell viability was assessed using the MTT assay. (J) A549 and DDP cells were treated with or without 3 μM CDDP for 48 hours, then harvested for Annexin V/PI staining. Annexin V-positive cells were quantified as apoptotic cells. At least three independent experiments were performed for each sample. (K) A549 and DDP cells were treated with different concentrations of CDDP for 48 hours, and then cells were harvested for IB against cleaved-PARP (C-PARP) to measure the level of apoptosis. (L) A549 cells were treated with various concentrations of IL-33 recombinant protein for 48 hours, after which the cells were harvested for IB.

**Supplementary Figure 3**

1. A549 cells were treated with 1 μM Dox in combination with or without 10 μM MAPK inhibitor SB 203580 for 48 hours, and then harvested for IB analysis. (B) IB quality control was conducted using virus-infected A549 cells before performing the CUT&Tag assay, including IL-33-F OE and IL-33-C OE groups. (C) Integrative Genomics Viewer of IL-33 full-length and IL-33 cytokine domain CUT&Tag signals for the XRCC2, BRCA2, RAD51 and RAD50 genes. (D) Enrichment of GO signatures for proteins regulated by IL-33-F OE. (E) GSEA of TMT quantitation data showing enrichment of the DNA repair complex gene signature in A549 cells. (F) Heat map analysis of DNA repair complex genes in A549 cells using TMT quantitation data. (G) Virus-infected A549 cells (Ctrl, IL-33-F OE, and IL-33-C OE) were harvested for qRT-PCR.

**Supplementary Figure 4**

1. IL-33 IF-IHC staining of tumor sections from each group. (B) γ-H2AX IF-IHC staining of tumor sections from each group. (C) Cleaved-Caspase 3 IF-IHC staining of tumor sections from each group. (D) Ki-67 IF-IHC staining of tumor sections from each group. Scale bar: 50 μm.

**Supplementary Figure 5**

(A) A549 cells, either in single culture or co-cultured with CAFs, were treated with 3 μM CDDP for 24 hours, and then cells were harvested for IB analysis. (B) CAFs were treated with or without 3 μM CDDP for 24 hours, and then cells were harvested for IB analysis. (C) CAFs, A549 cells, and A549 cells co-cultured with CAFs were treated with or without 3 μM CDDP for 24 hours, and the cell culture supernatants were collected for ELISA.

**Supplementary Figure 6**

Proposed model illustrating how IL-33 promotes DNA damage repair to limit the efficacy of chemotherapy in lung cancer. IL-33, derived from both CAFs and the tumor itself, binds to the ST2 receptor on lung cancer cells. This interaction activates downstream MAPK pathways and enhances DNA repair through the NHEJ pathway. Additionally, full length IL-33 can also function as a transcription factor in the cell nucleus, promoting the expression of genes associated with the HR pathway, further facilitating the repair of damaged DNA and DNA damage tolerance.
