# Supplementary material for: Interleukin-33 (IL-33) promotes DNA damage-resistance in lung cancer
Source: Cell Death Dis. 2025 Apr 11;16(1):274. doi: 10.1038/s41419-025-07624-x (PMC11992107; doi:10.1038/s41419-025-07624-x)
Supplement: Supplementary file 3 — Supplemental figures [file 41419_2025_7624_MOESM3_ESM.pdf]

Figure S1

Summary | Gene Expression

4,511
Number of Spots Under Tissue

49,851	3,391
Mean Reads per Spot	Median Genes per Spot

Sequencing

Number of Reads	224,878,869
Valid Barcodes	99.1%
Valid UMIs	99.8%
Sequencing Saturation	74.4%
Q30 Bases in Barcode	96.7%
Q30 Bases in Probe Read	97.6%
Q30 Bases in UMI	97.1%

Mapping

Reads Mapped to Probe Set	99.0%
Reads Mapped Confidently to Probe Set	98.7%
Reads Mapped Confidently to the Filtered Probe Set	96.4%

Image

Tissue Detection and Fiducial Alignment

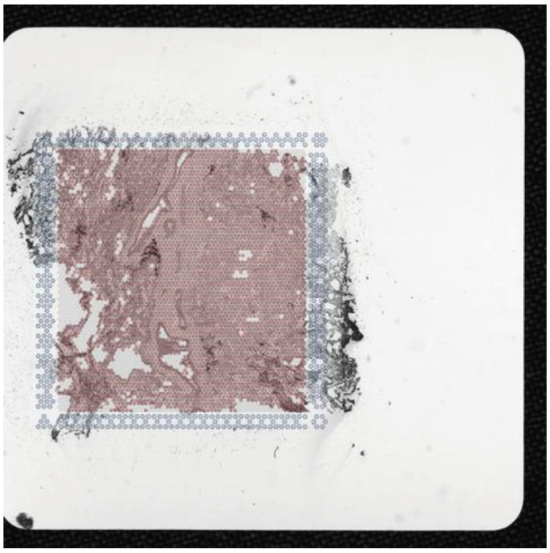

CytAssist Image Alignment

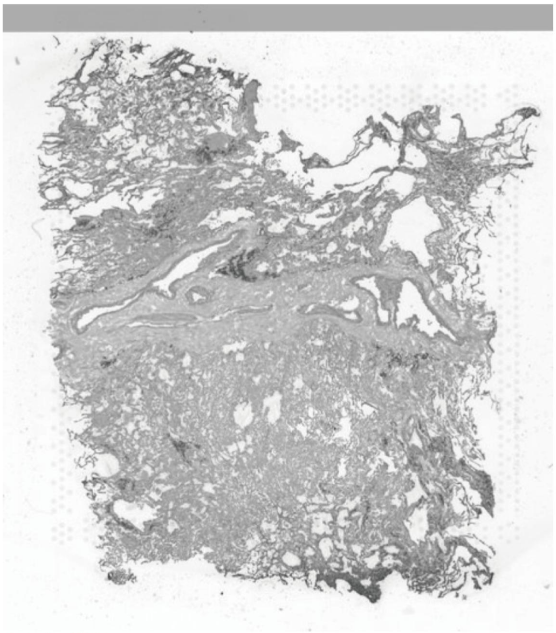

Opacity Slider

Spots

Fraction Reads in Spots Under Tissue	96.7%
Mean Reads per Spot	49,851
Mean Reads Under Tissue per Spot	47,745
Median UMI Counts per Spot	8,200
Median Genes per Spot	3,391
Genes Detected	16,516

Figure S2

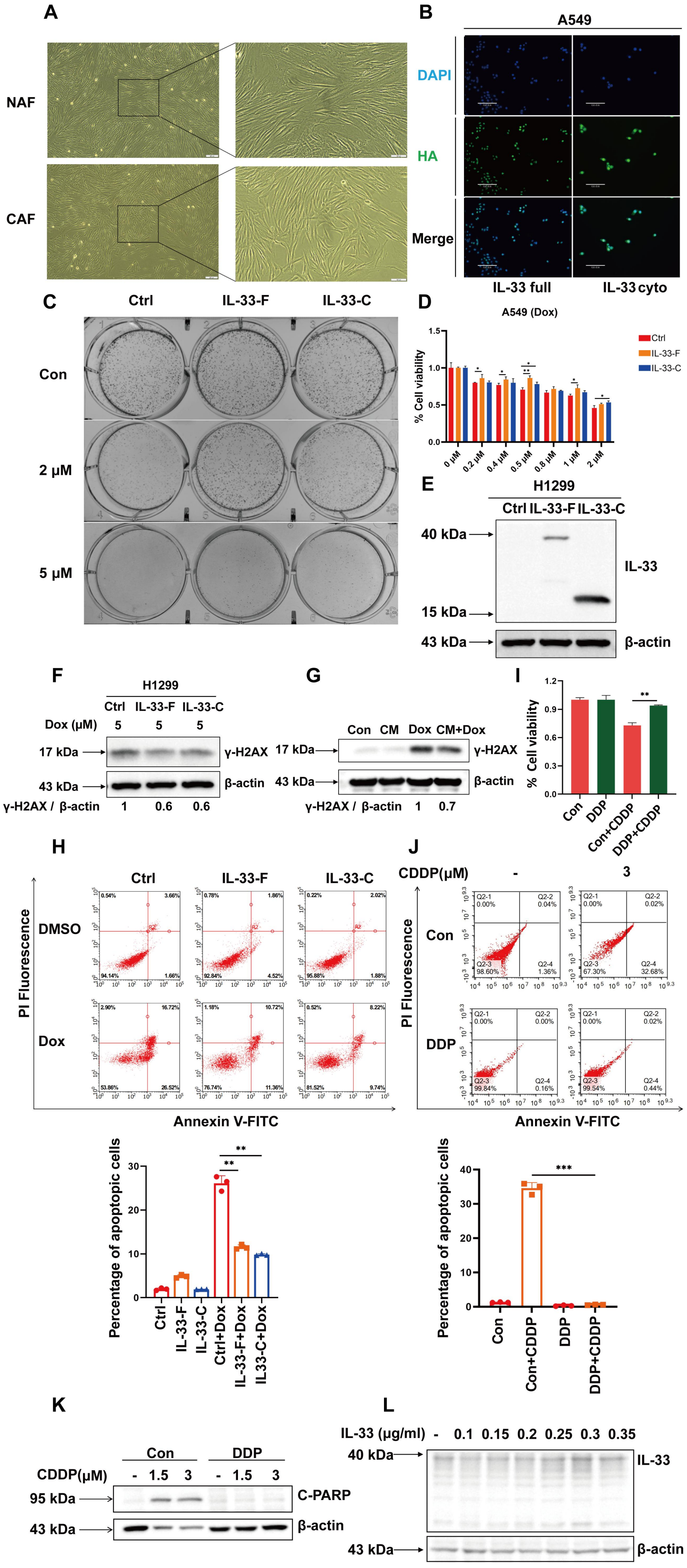
A
NAF
CAF
B
A549
DAPI
HA
Merge
IL-33 full
IL-33 cyto
C
Ctrl
IL-33-F
IL-33-C
Con
2 μM
5 μM
D
A549 (Dox)
% Cell viability
Ctrl
IL-33-F
IL-33-C
0 μM
0.2 μM
0.4 μM
0.5 μM
0.8 μM
1 μM
2 μM
E
H1299
Ctrl IL-33-F IL-33-C
40 kDa
15 kDa
43 kDa
IL-33
β-actin
F
H1299
Ctrl IL-33-F IL-33-C
Dox (μM) 5 5 5
17 kDa
43 kDa
γ-H2AX
β-actin
γ-H2AX / β-actin 1 0.6 0.6
G
Con CM Dox CM+Dox
17 kDa
43 kDa
γ-H2AX
β-actin
γ-H2AX / β-actin 1 0.7
I
% Cell viability
Con
DDP
Con+CDDP
DDP+CDDP
H
Ctrl
IL-33-F
IL-33-C
DMSO
Dox
PI Fluorescence
Annexin V-FITC
J
CDDP(μM)
-
3
Con
DDP
PI Fluorescence
Annexin V-FITC
Percentage of apoptopic cells
Ctrl
IL-33-F
IL-33-C
Ctrl+Dox
IL-33-F+Dox
IL33-C+Dox
Percentage of apoptopic cells
Con
Con+CDDP
DDP
DDP+CDDP
K
Con
DDP
CDDP(μM) - 1.5 3 - 1.5 3
95 kDa
43 kDa
C-PARP
β-actin
L
IL-33 (μg/ml) - 0.1 0.15 0.2 0.25 0.3 0.35
40 kDa
43 kDa
IL-33
β-actin

Figure S3

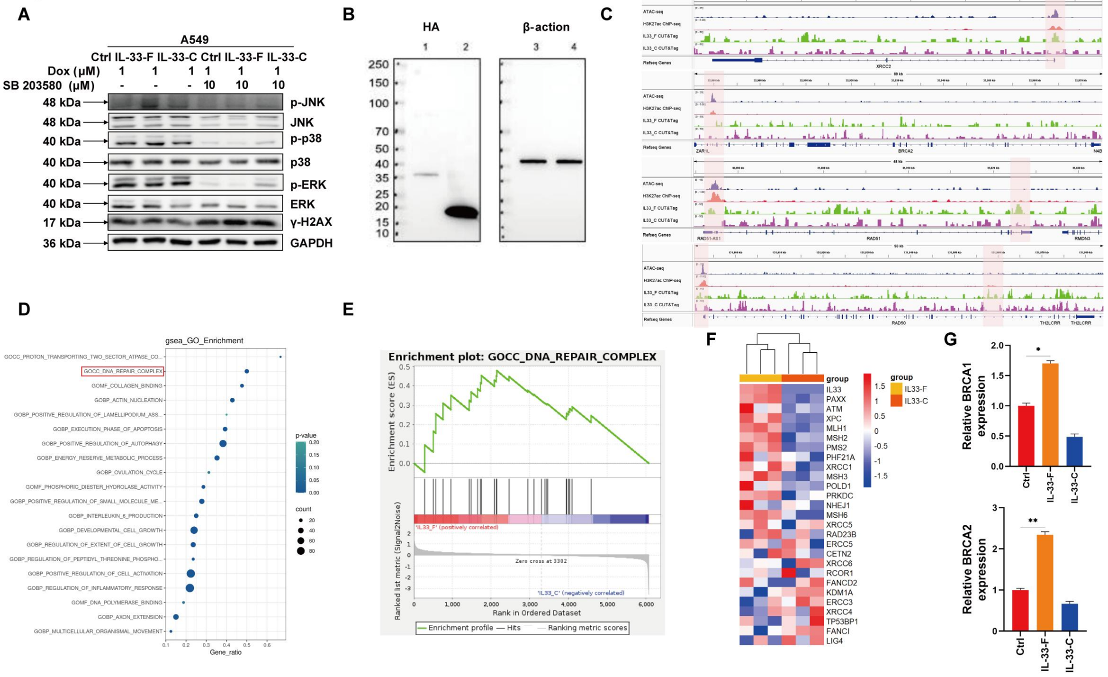
A
A549
Ctrl IL-33-F IL-33-C Ctrl IL-33-F IL-33-C
Dox (μM) 1 1 1 1 1 1
SB 203580 (μM) - - - 10 10 10
48 kDa p-JNK
48 kDa JNK
40 kDa p-p38
40 kDa p38
40 kDa p-ERK
40 kDa ERK
17 kDa γ-H2AX
36 kDa GAPDH
B
HA
β-action
1 2 3 4
250 150 100 70 50 40 35 25 20 15 10
C
ATAC-seq
H3K27ac ChIP-seq
IL33_F CUT&Tag
IL33_C CUT&Tag
Refseq Genes
XRCC3
BRCA2
RAD51
RAD50
D
gsea_GO_Enrichment
GOCC_PROTON_TRANSPORTING_TWO_SECTOR_ATPASE_CO...
GOCC_DNA_REPAIR_COMPLEX
GOMF_COLLAGEN_BINDING
GOBP_ACTIN_NUCLEATION
GOBP_POSITIVE_REGULATION_OF_LAMELLIPODIUM_ASS...
GOBP_EXECUTION_PHASE_OF_APOPTOSIS
GOBP_POSITIVE_REGULATION_OF_AUTOPHAGY
GOBP_ENERGY_RESERVE_METABOLIC_PROCESS
GOBP_OVULATION_CYCLE
GOMF_PHOSPHORIC_DIESTER_HYDROLASE_ACTIVITY
GOBP_POSITIVE_REGULATION_OF_SMALL_MOLECULE_ME...
GOBP_INTERLEUKIN_6_PRODUCTION
GOBP_DEVELOPMENTAL_CELL_GROWTH
GOBP_REGULATION_OF_EXTENT_OF_CELL_GROWTH
GOBP_REGULATION_OF_PEPTIDYL_THREONINE_PHOSPHO...
GOBP_POSITIVE_REGULATION_OF_CELL_ACTIVATION
GOBP_REGULATION_OF_INFLAMMATORY_RESPONSE
GOMF_DNA_POLYMERASE_BINDING
GOBP_AXON_EXTENSION
GOBP_MULTICELLULAR_ORGANISMAL_MOVEMENT
p-value
count
Gene_ratio
E
Enrichment plot: GOCC_DNA_REPAIR_COMPLEX
Enrichment score (ES)
'IL33_F' (positively correlated)
Zero cross at 3382
'IL33_C' (negatively correlated)
Ranked list metric (Signal2Noise)
Rank in Ordered Dataset
Enrichment profile — Hits — Ranking metric scores
F
group
IL33-F
IL33-C
IL33
PAXX
ATM
XPC
MLH1
MSH2
PMS2
PHF21A
XRCC1
MSH3
POLD1
PRKDC
NHEJ1
MSH6
XRCC5
RAD23B
ERCC5
CETN2
XRCC6
RCOR1
FANCD2
KDM1A
ERCC3
XRCC4
TP53BP1
FANCI
LIG4
G
Relative BRCA1 expression
Relative BRCA2 expression
Ctrl
IL-33-F
IL-33-C

Figure S4

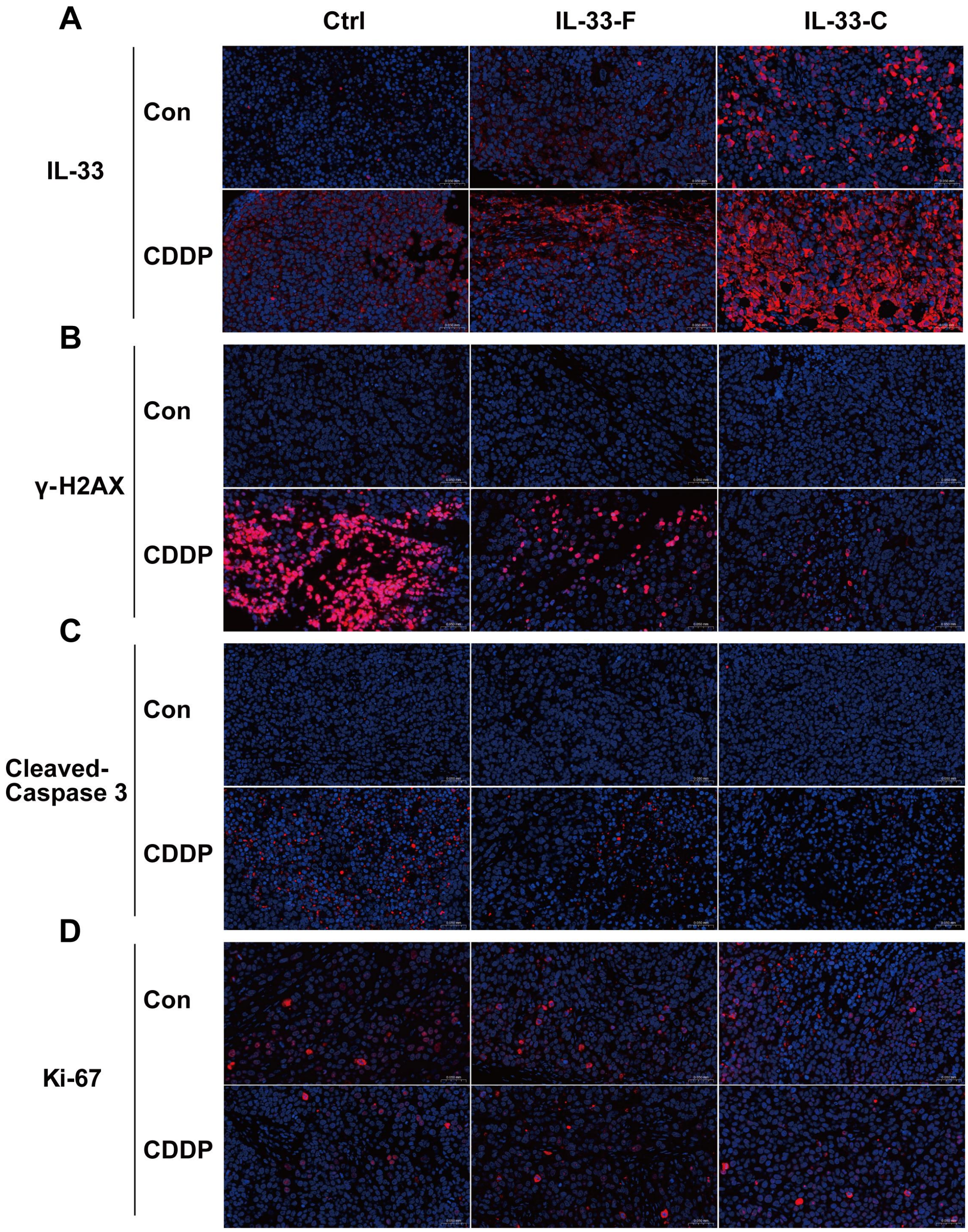
A
B
C
D
Ctrl
IL-33-F
IL-33-C
IL-33
γ-H2AX
Cleaved-Caspase 3
Ki-67
Con
CDDP

Figure S5

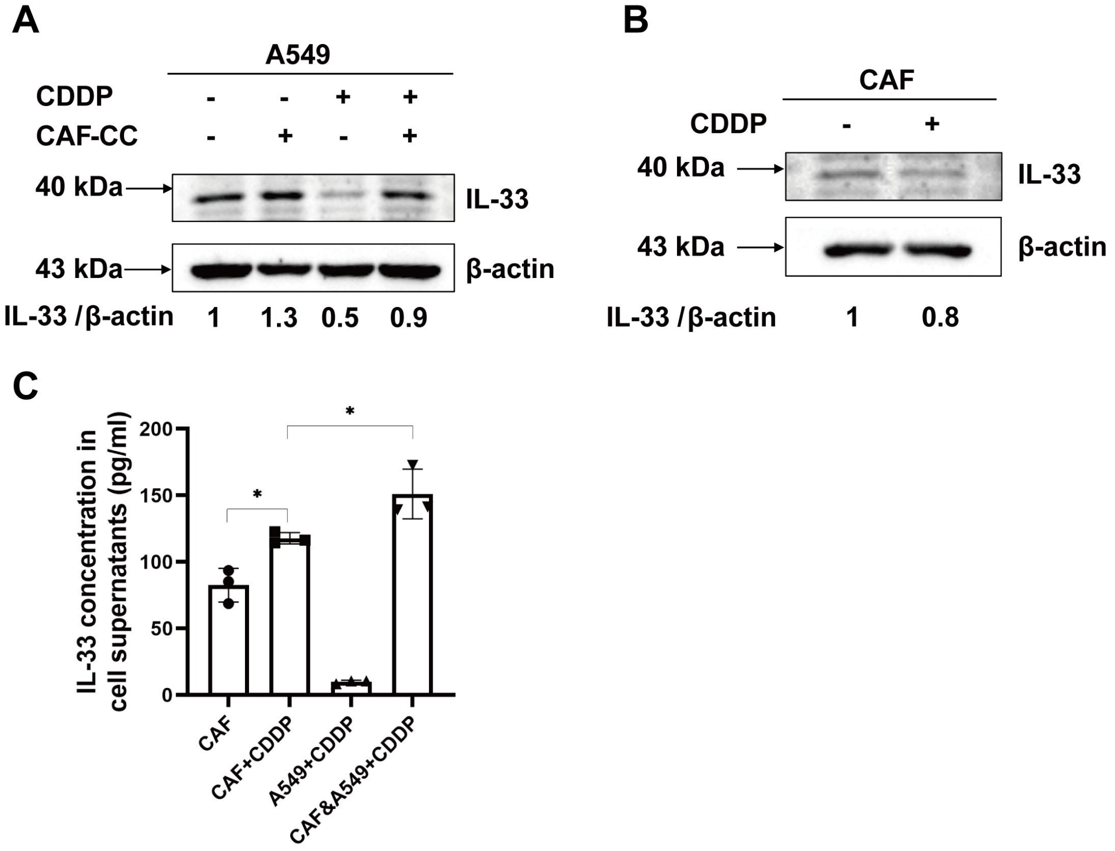
A
A549
CDDP - - + +
CAF-CC - + - +
40 kDa
IL-33
43 kDa
β-actin
IL-33 /β-actin 1 1.3 0.5 0.9
B
CAF
CDDP - +
40 kDa
IL-33
43 kDa
β-actin
IL-33 /β-actin 1 0.8
C
IL-33 concentration in cell supernatants (pg/ml)
200
150
100
50
0
*
*
CAF
CAF+CDDP
A549+CDDP
CAF&A549+CDDP

Figure S6

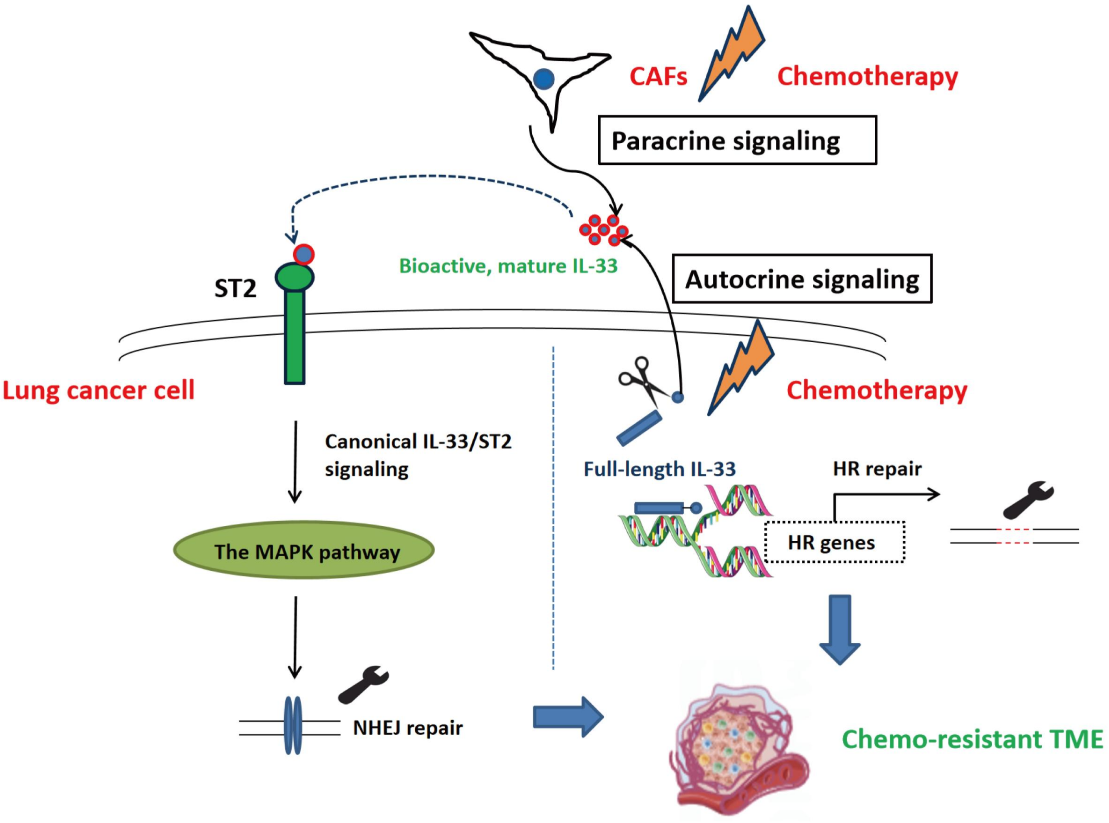

CAFs
Chemotherapy
Paracrine signaling
Bioactive, mature IL-33
Autocrine signaling
ST2
Lung cancer cell
Chemotherapy
Canonical IL-33/ST2 signaling
Full-length IL-33
HR repair
HR genes
The MAPK pathway
NHEJ repair
Chemo-resistant TME
