# Supplementary material for: Interleukin-33 (IL-33) promotes DNA damage-resistance in lung cancer
Source: Cell Death Dis. 2025 Apr 11;16(1):274. doi: 10.1038/s41419-025-07624-x (PMC11992107; doi:10.1038/s41419-025-07624-x)

**Original images for Figure 2 and Figure3**


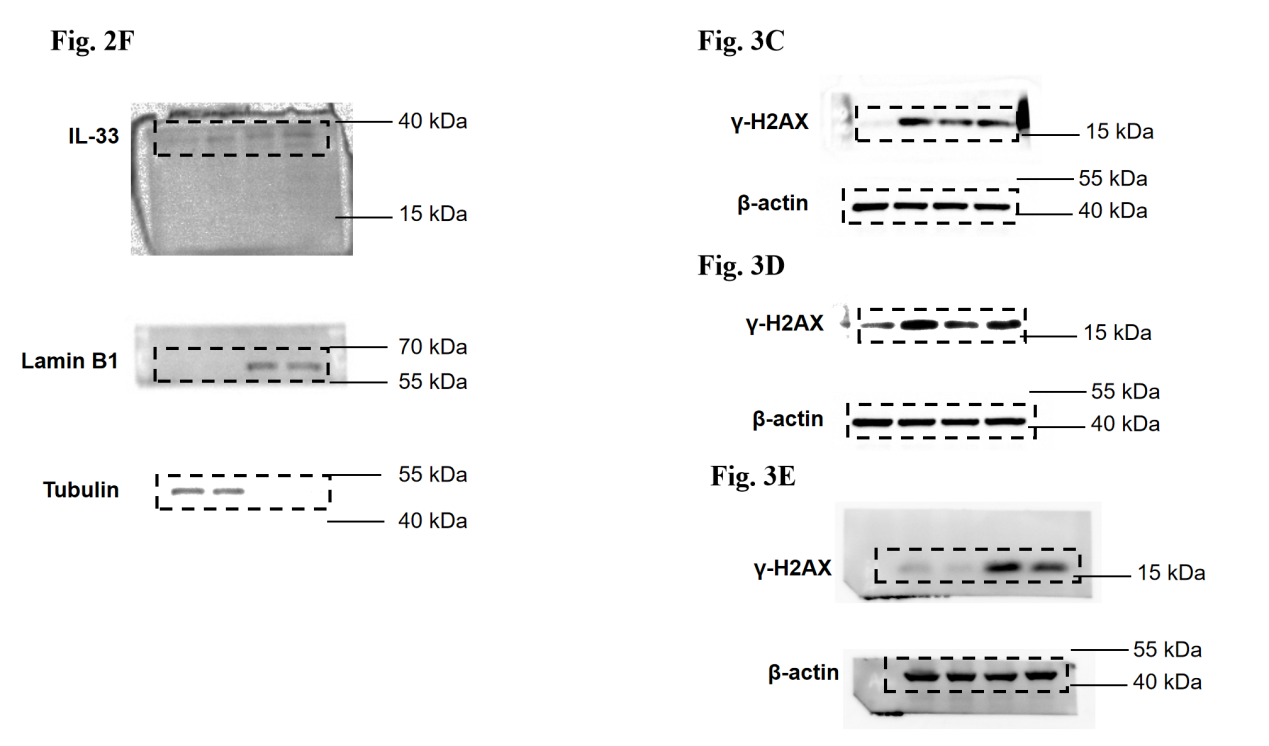


**Original images for Figure 4**


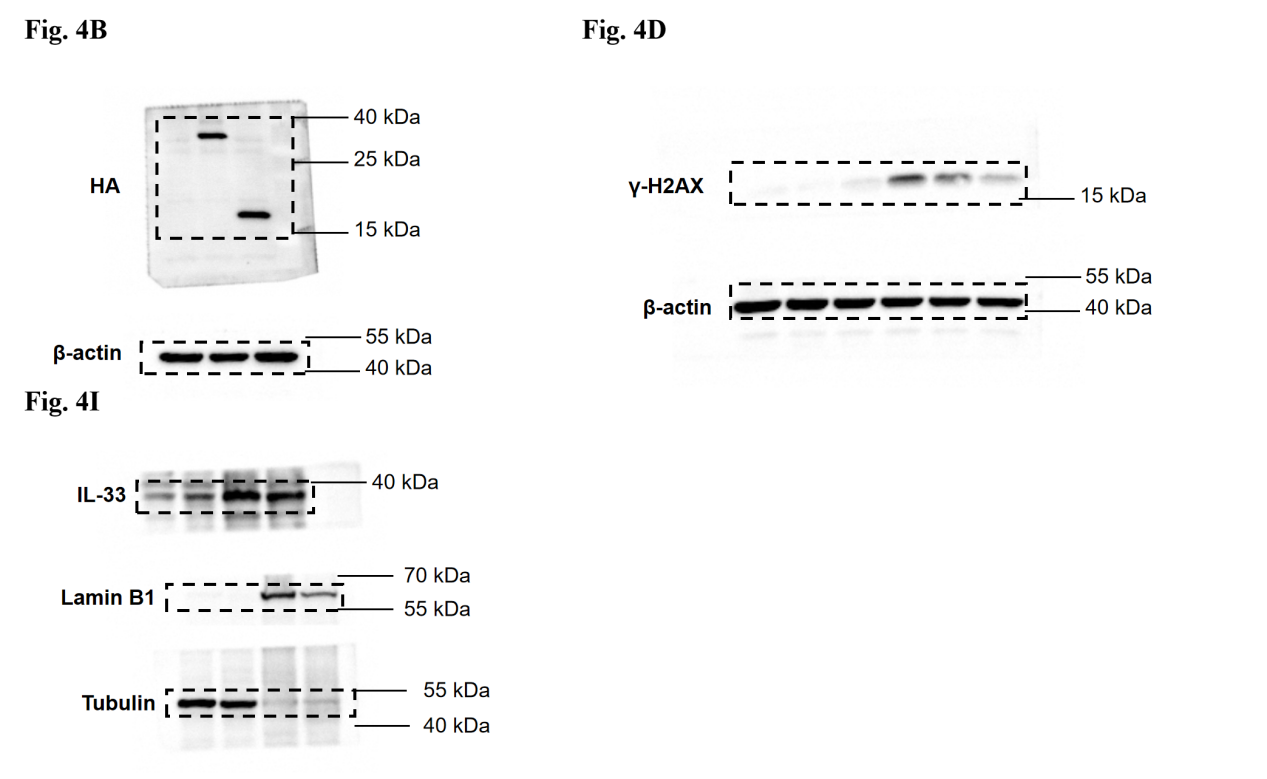


**Original images for Figure 5**


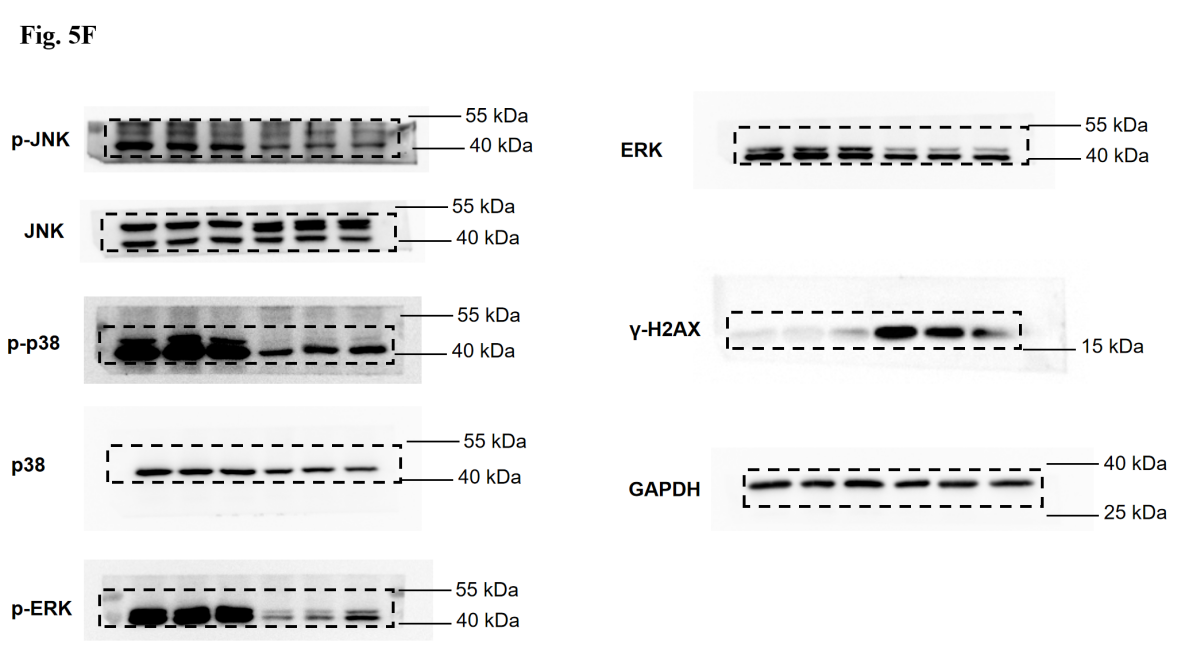


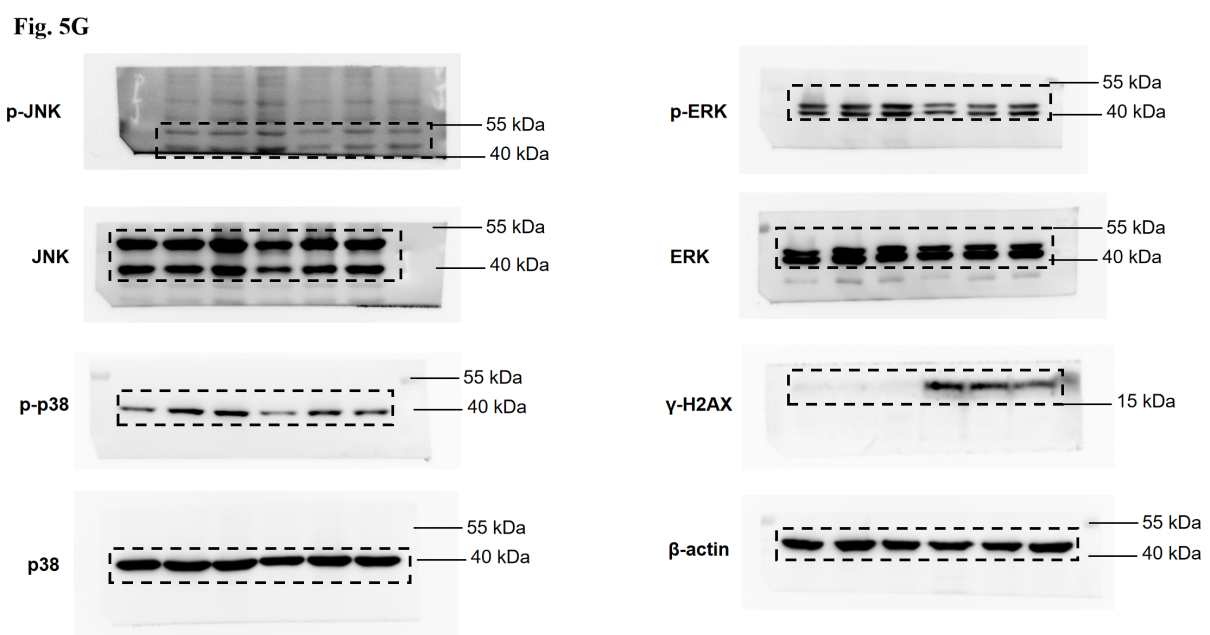


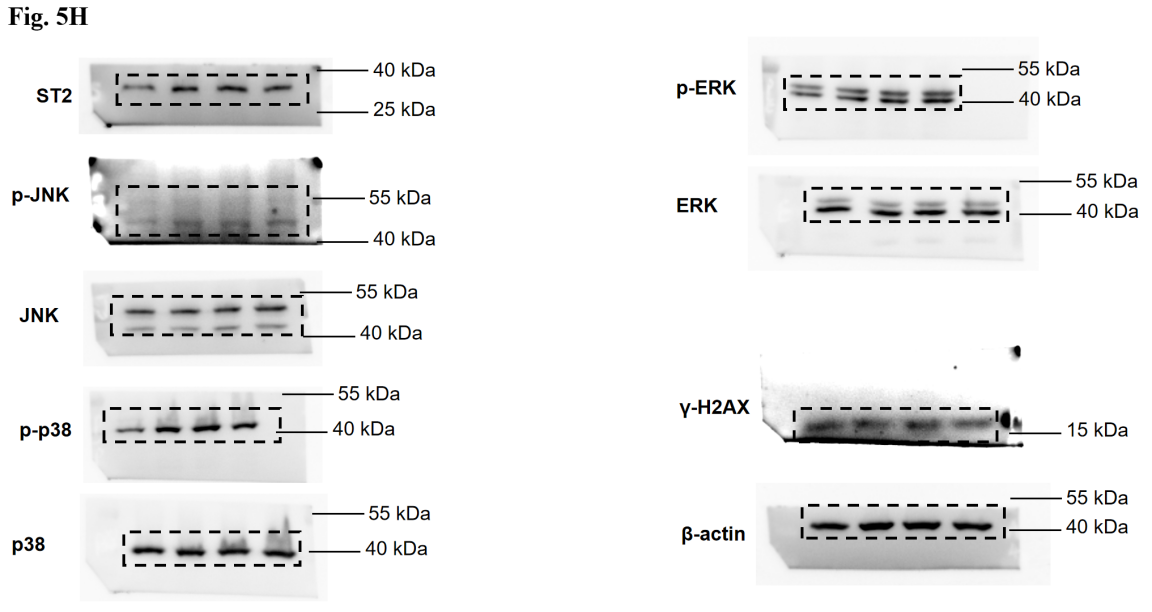


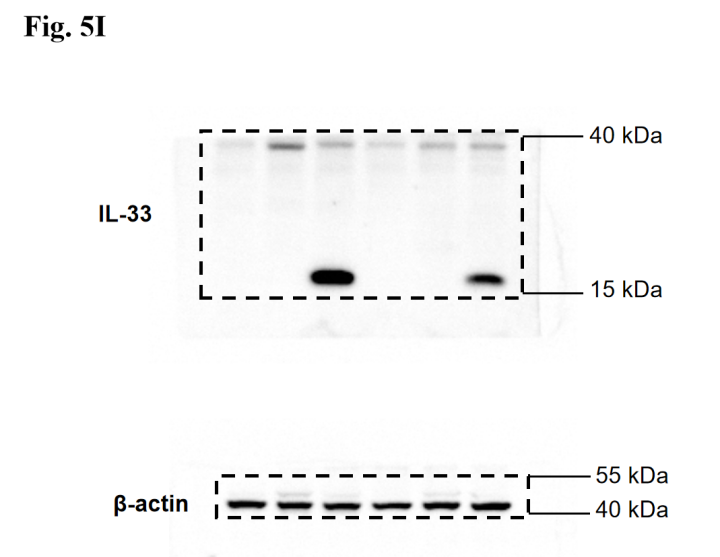


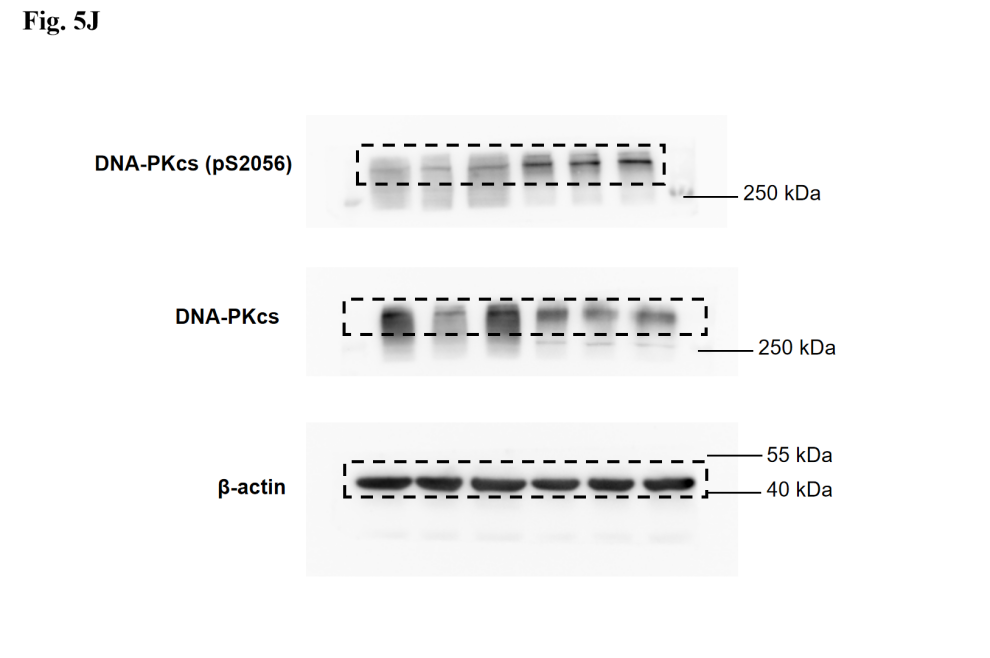


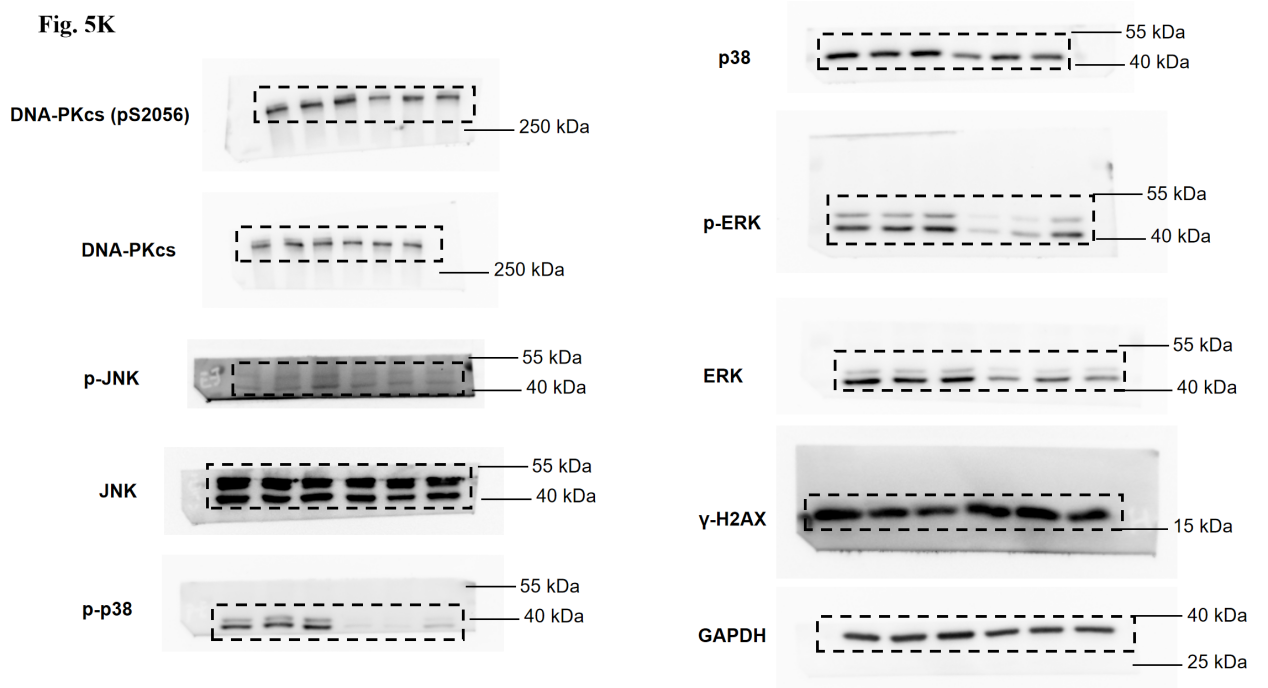


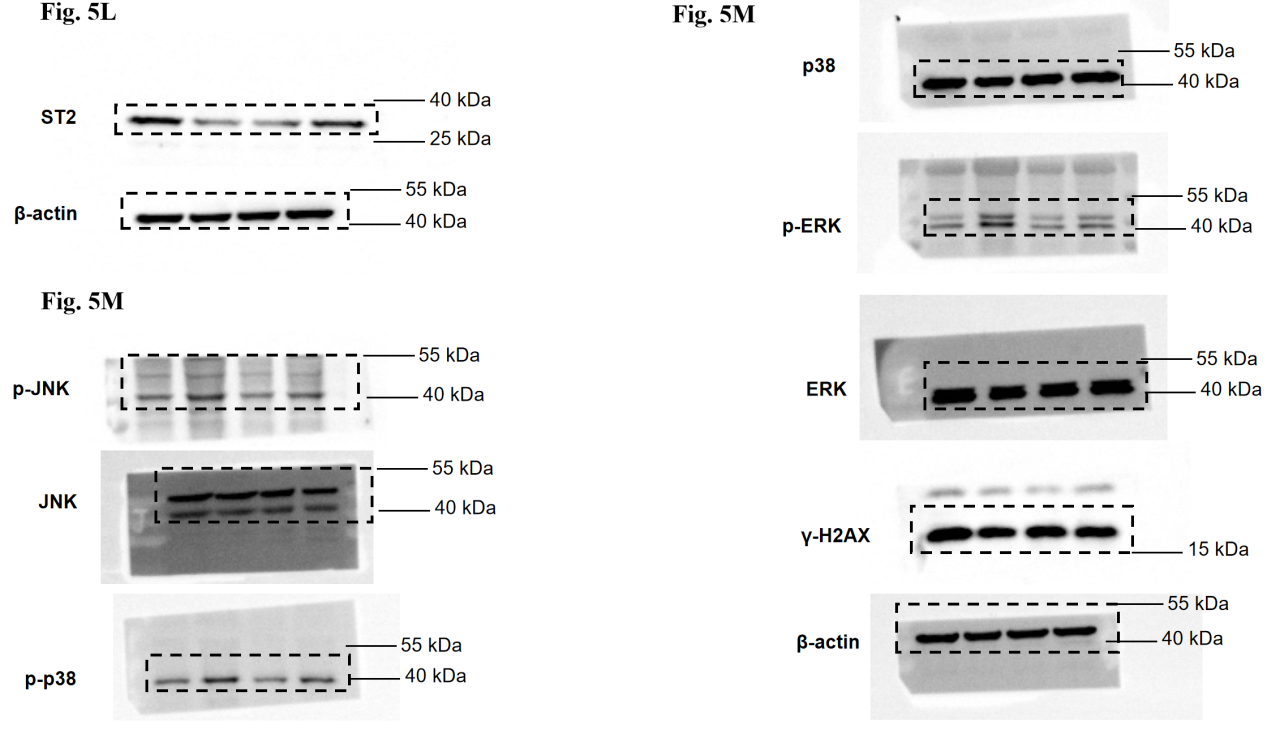


**Original images for Figure 6**


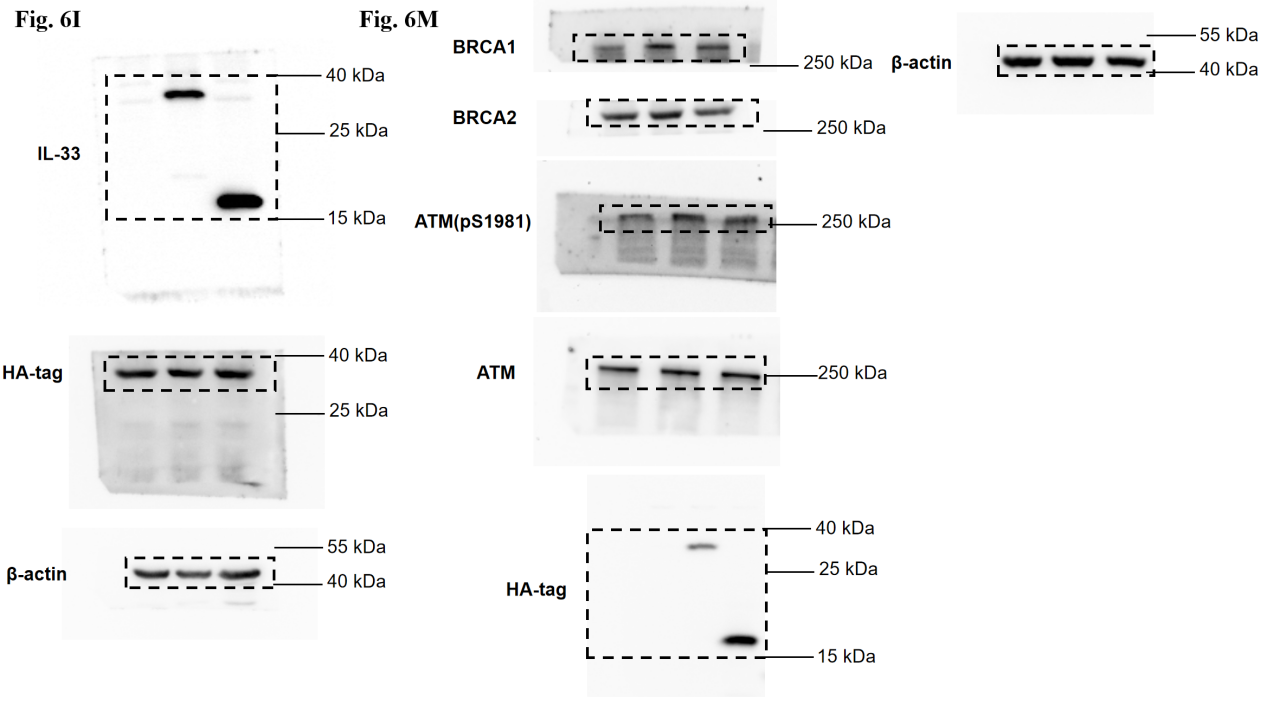


**Original images for Figure S2**


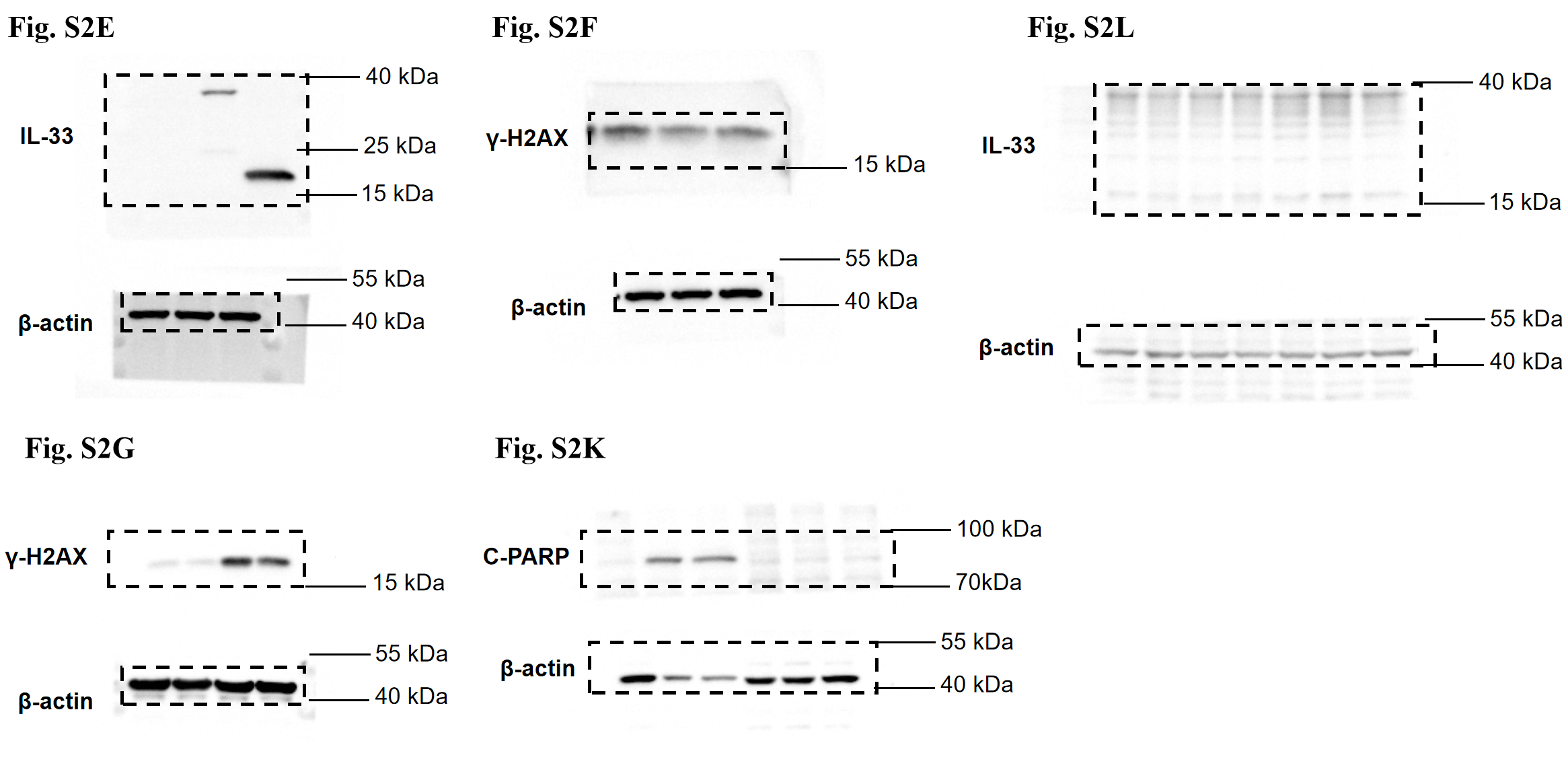


**Original images for Figure S3**


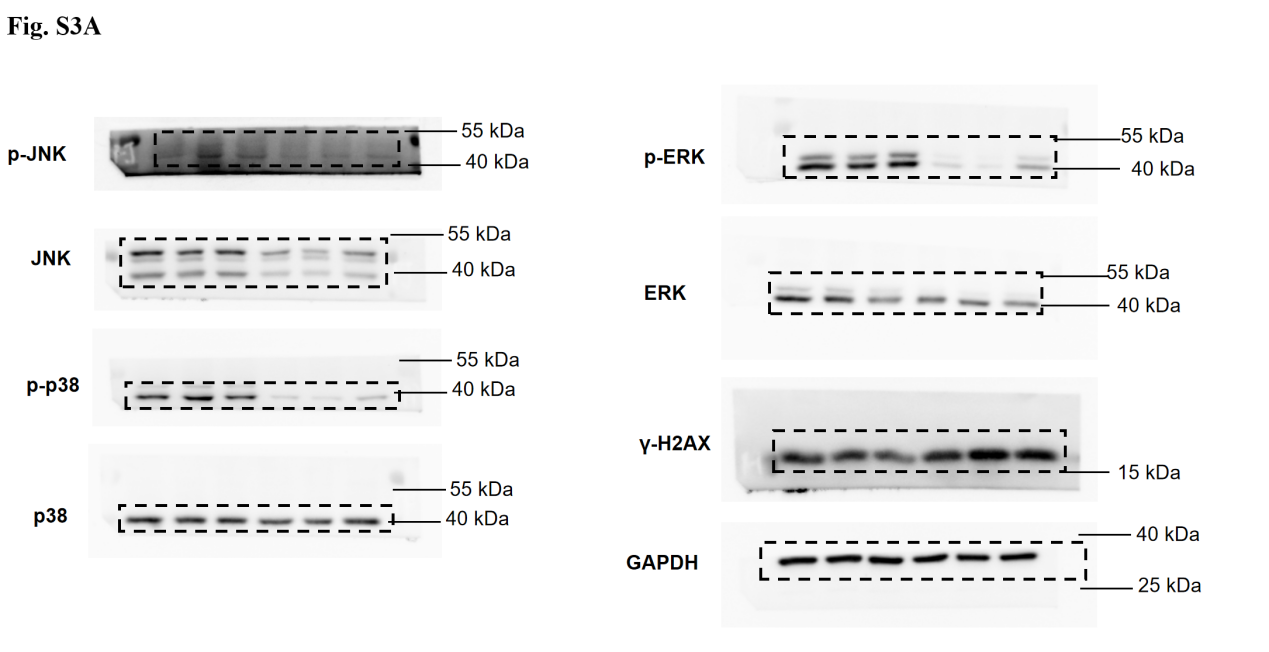


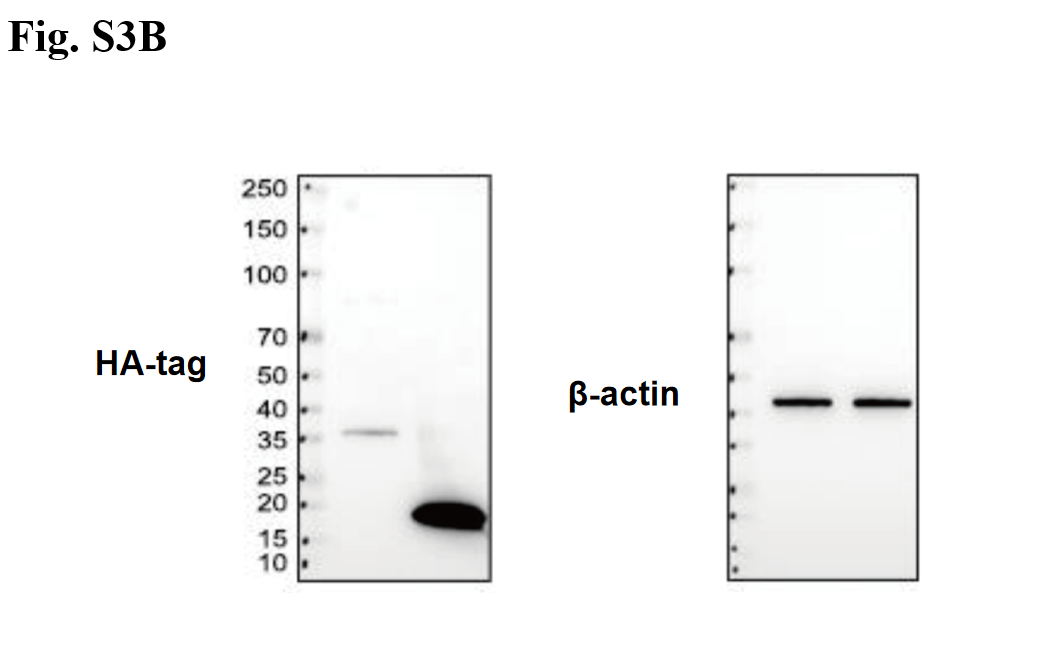


**Original images for Figure S5**


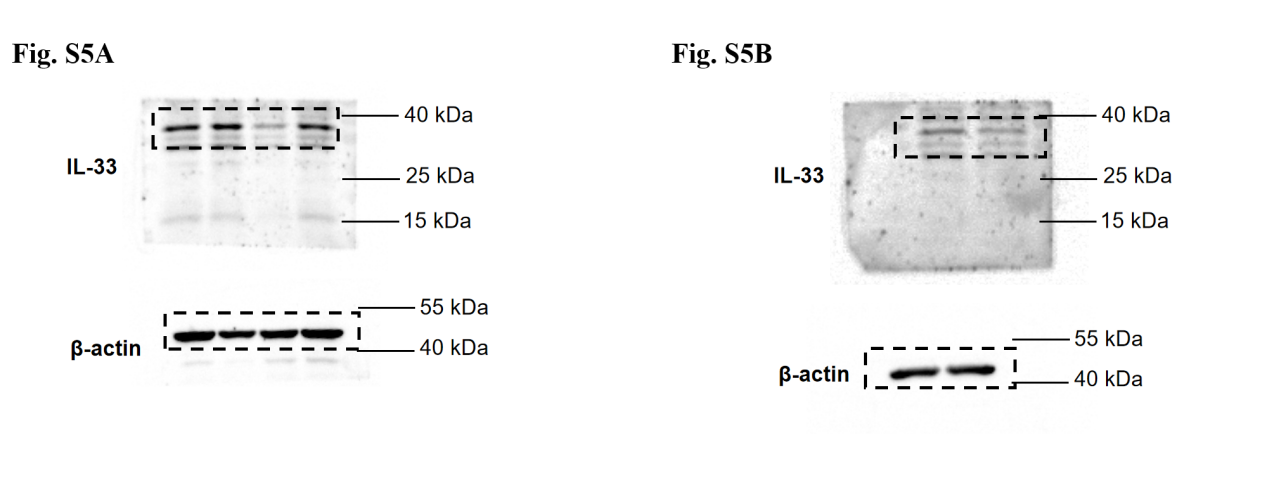

Supplement: Supplementary file 5 — Full western blots images [file 41419_2025_7624_MOESM5_ESM.docx]
